# Evaluating atmospheric mercury (Hg) uptake by vegetation in a chemistry-transport model†

**DOI:** 10.1039/d2em00032f

**Published:** 2022-04-22

**Authors:** Aryeh Feinberg, Thandolwethu Dlamini, Martin Jiskra, Viral Shah, Noelle E. Selin

**Affiliations:** Institute for Data, Systems, and Society, Massachusetts Institute of Technology Cambridge MA USA arifein@mit.edu selin@mit.edu; Environmental Geosciences, University of Basel Basel Switzerland; Harvard John A. Paulson School of Engineering and Applied Sciences, Harvard University Cambridge MA USA; Department of Earth, Atmospheric, and Planetary Sciences, Massachusetts Institute of Technology Cambridge MA USA

## Abstract

Mercury (Hg), a neurotoxic heavy metal, is transferred to marine and terrestrial ecosystems through atmospheric transport. Recent studies have highlighted the role of vegetation uptake as a sink for atmospheric elemental mercury (Hg^0^) and a source of Hg to soils. However, the global magnitude of the Hg^0^ vegetation uptake flux is highly uncertain, with estimates ranging 1000–4000 Mg per year. To constrain this sink, we compare simulations in the chemical transport model GEOS-Chem with a compiled database of litterfall, throughfall, and flux tower measurements from 93 forested sites. The prior version of GEOS-Chem predicts median Hg^0^ dry deposition velocities similar to litterfall measurements from Northern hemisphere temperate and boreal forests (∼0.03 cm s^−1^), yet it underestimates measurements from a flux tower study (0.04 cm s^−1^*vs.* 0.07 cm s^−1^) and Amazon litterfall (0.05 cm s^−1^*vs.* 0.17 cm s^−1^). After revising the Hg^0^ reactivity within the dry deposition parametrization to match flux tower and Amazon measurements, GEOS-Chem displays improved agreement with the seasonality of atmospheric Hg^0^ observations in the Northern midlatitudes. Additionally, the modelled bias in Hg^0^ concentrations in South America decreases from +0.21 ng m^−3^ to +0.05 ng m^−3^. We calculate a global flux of Hg^0^ dry deposition to land of 2276 Mg per year, approximately double previous model estimates. The Amazon rainforest contributes 29% of the total Hg^0^ land sink, yet continued deforestation and climate change threatens the rainforest's stability and thus its role as an important Hg sink. In an illustrative worst-case scenario where the Amazon is completely converted to savannah, GEOS-Chem predicts that an additional 283 Mg Hg per year would deposit to the ocean, where it can bioaccumulate in the marine food chain. Biosphere–atmosphere interactions thus play a crucial role in global Hg cycling and should be considered in assessments of future Hg pollution.

## Abstract 2

We study the uptake of atmospheric mercury by vegetation in a chemical transport model and available observations. Due to the importance of this sink in the global mercury cycle, perturbations to forested areas can elevate mercury risks.

## Graphical abstract

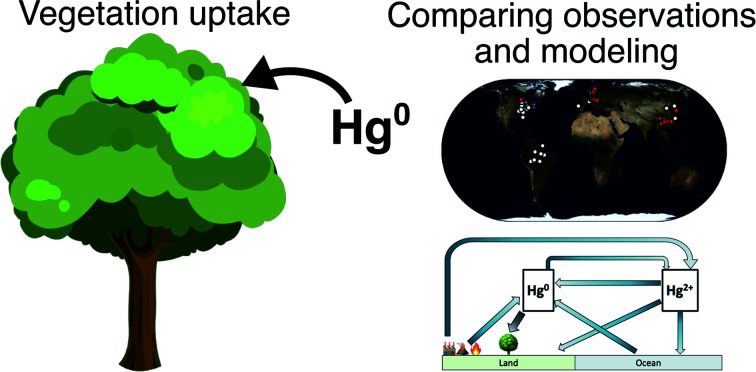

**Environmental significance**

Vegetation uptake is one of largest sinks of atmospheric mercury (Hg) from the atmosphere and a major source of Hg to soils. We better quantify its importance to the global biogeochemical Hg cycle by updating an atmospheric chemistry model with information from newly available measurement datasets. Our revised dry deposition scheme yields improved model agreement with atmospheric Hg seasonality in Northern midlatitudes and Hg concentrations in South America. We calculate a dry deposition flux to land that is approximately double previous model estimates. Using our revised model, we also illustrate the potential importance of land-atmosphere feedbacks: the conversion of the Amazon rainforest to savannah leads to an additional transfer of 283 Mg Hg each year to the ocean due to a reduced land sink.

## 1. Introduction

Mercury (Hg) is a neurotoxic heavy metal that bioaccumulates in marine and terrestrial food webs as methylmercury (MeHg). Approximately 8000 Mg per year of Hg are emitted to the atmosphere each year, of which 30% are emitted directly by anthropogenic sources, 10% are from natural geogenic sources, and 60% are re-emissions of anthropogenic Hg that was previously deposited to land and water.^1^ Atmospheric Hg is transported to remote regions due to its long lifetime of around 6 months before being ultimately removed by wet and dry deposition.^2,3^ Recent studies have emphasized the role that vegetation plays in the dry deposition of elemental mercury (Hg^0^), with estimates of this sink ranging from 1000 to 4046 Mg per year.^4–6^ Isotopic evidence suggests that atmospheric Hg^0^ deposition is the source of 57–94% of all Hg in soils.^5^ Due to this link between atmospheric Hg and the biosphere, atmospheric Hg levels can be altered by seasonality and trends in vegetation productivity.^4^ Climate change and anthropogenic activities could disturb the Hg vegetation sink through multiple processes, *e.g.*, deforestation,^7^ CO_2_ fertilization,^8^ vegetation species shifts,^9^ drought,^10^ and ice storms.^11^ In particular, there is a risk that the Amazon, the largest rainforest on the planet, could transition into a savannah-like ecosystem due to anthropogenic pressures (*i.e.* deforestation) and climate change.^12–14^ An accurate representation of Hg^0^ vegetation uptake in the global Hg budget is essential for predicting the impact of these perturbations on Hg cycling. However, until now there has been only narrow validation of atmospheric Hg chemistry models with observations of vegetation uptake, mainly focusing on selected sites in North America.^9,15–17^

Almost all atmospheric Hg chemistry models (including GEOS-Chem, ECHMERIT, GEM-MACH-Hg, and GLEMOS) use a resistance-based dry deposition scheme.^15,18^ In these schemes, canopy uptake processes are parametrized as a function of leaf area index (LAI) and land cover type.^19,20^ There is considerable uncertainty associated with the model parameters related to Hg dry deposition.^5^ The evaluation of model parametrizations is complicated by the systematic biases inherent to different experimental methods quantifying the uptake of Hg by vegetation (Table 1). Litterfall measurements are used to determine the amount of Hg taken up by foliage over a growing season, which isotopic evidence suggests mainly originate from atmospheric Hg^0^ uptake as opposed to oxidized mercury (Hg^2+^).^21–23^ Measurements of throughfall (water falling through the forest canopy) capture another portion of dry deposited Hg, washing off Hg adsorbed to canopy surfaces.^24^ Isotope evidence suggests that 34% to 82% of Hg in throughfall is derived from adsorbed atmospheric Hg^0^.^25^ However, litterfall and throughfall measurements do not account for uptake of Hg by woody tissues, mosses, and lichens in forest ecosystems.^6,25^ Whole ecosystem net Hg^0^ exchange has been measured directly by micrometeorological methods, which employ tower measurements and flux-gradient calculations.^24,26^ Micrometeorological techniques also measure net ecosystem exchange fluxes at higher time resolutions (30 min) than throughfall and litterfall measurements. Despite being the more accurate technique, there is currently only one annual micrometeorological measurement from a temperate deciduous forest site^26^ to compare with atmospheric model predictions.

**Table 1:** Review of measurement methods quantifying Hg vegetation uptake, compared in this study with GEOS-Chem simulated Hg^0^ dry deposition in forested areas

Name	Description	Species of Hg	Potential biases when comparing with modelled Hg^0^ dry deposition
Litterfall	Collecting and analyzing plant litter to measure net Hg uptake in leaves over the growing season	Hg^0^^a^	Missing uptake from woody tissues, lichens, mosses, and soil; missing Hg^0^ contribution from throughfall; foliar re-emission not explicitly modelled
Throughfall	Measuring flux of Hg washed off leaves during precipitation events with a collector placed under the canopy	Hg^0^, Hg^2+^^b^	—c
Total foliar uptake	Calculated as litterfall + throughfall − open field wet deposition	Hg^0^, Hg^2+^	Missing uptake from woody tissues, lichens, mosses, and soil; includes Hg^2+^ contribution from throughfall; foliar re-emission not explicitly modelled
Micrometeorological flux tower methods	Using Hg^0^ tower measurements and flux-gradient calculations to calculate whole net-ecosystem exchange	Hg^0^	Only one annual measurement available in forested areas;^26^ if soil emissions are high, net-ecosystem exchange underestimates Hg^0^ vegetation uptake; foliar re-emission not explicitly modelled

a Demers *et al.*;^21^ Jiskra *et al.*;^22^ Zheng *et al.*^23^

b Wang *et al.*^25^

c Throughfall is not directly compared to modelled Hg^0^ dry deposition.

Past studies have calculated a broad range of magnitudes for the global Hg vegetation sink, depending on the monitoring data and scaling approach used. Wang *et al.*^27^ estimated that 1180 ± 710 Mg Hg per year deposits as litterfall, using a compilation of litterfall data and statistical modeling. Total throughfall has been estimated to contribute 1338 Mg per year over forests, which includes both wet deposition and dry-deposited Hg washed off leaves.^28^ By extrapolating flux tower measurements from Harvard Forest, Obrist *et al.*^26^ calculated a global Hg^0^ uptake flux of 3162–3813 Mg per year, approximately a factor of three higher than the litterfall assessment.^27^ A new global database approach, considering other vegetation components in addition to litterfall, also reported a larger estimate for the global vegetation uptake flux: 2705 ± 504 Mg per year.^6^ It remains unclear how these larger fluxes can be accommodated within the global Hg budget and whether they are compatible with other observational constraints in the Hg cycle, *e.g.*, atmospheric concentrations, wet deposition fluxes, and isotopic tracers.^29^ Global atmospheric models like GEOS-Chem are useful tools for evaluating these broader constraints on the global Hg budget. However, recent work suggests that global atmospheric models underestimate vegetation uptake compared to tropical litterfall data^5^ and net exchange fluxes measured over a midlatitude deciduous forest.^26^

In this study, we amend the Hg^0^ dry deposition scheme in GEOS-Chem to integrate information derived from a broad dataset of available vegetation uptake measurements. We focus our comparison between model and measurements in forested sites, since forests are expected to have the largest contribution to vegetation uptake.^30^ By adjusting the biological reactivity of Hg^0^ in the dry deposition scheme, we propose model configurations that are compatible with different vegetation uptake measurement methods (litterfall; total foliar uptake; micrometeorological net Hg^0^ exchange, see Table 1). We evaluate the impacts of these adjustments on the modelled atmospheric Hg seasonality and spatial distribution. Using the model configuration that best matches available observational constraints, we provide improved estimates of the global magnitude of the vegetation sink and other Hg budget terms. We explore the effect of an extreme scenario for Amazon land cover change on the fate of atmospheric Hg and discuss its implications for future Hg cycling. Finally, we discuss future directions for the development of model parametrizations of Hg vegetation uptake.

## 2. Materials and methods

### 2.1 Compilation of litterfall, throughfall, and dry deposition measurements

To evaluate the performance of the GEOS-Chem dry deposition scheme in simulating Hg^0^ dry deposition over forests, we compiled an observational database of vegetation uptake measurements for forested areas. We focused on studies which measured litterfall, throughfall, and/or open field wet deposition. For study sites where all three quantities were measured, we calculated a total foliar uptake flux = litterfall + throughfall − open field wet deposition, which represents the total dry deposited Hg to foliage.^24^ In addition, we evaluate the model against net ecosystem exchange flux of Hg^0^ at a temperate deciduous hardwood forest site.^26^

We used previous reviews of Hg dry deposition as starting points for the database.^24,31,32^ To identify more recent litterfall studies, we screened 334 results from a Web of Science (Clarivate Analytics) search with the term “mercury litter*“. In total, the database contains 79 publications with measurement-based estimates of Hg uptake fluxes. We extracted additional metadata from the compiled publications: geographic coordinates, altitude, year of study, and land cover type (ESI† Spreadsheet).

The database ranges temporally from 1987 to 2020. Regional trends in atmospheric Hg^0^ concentrations also affect the magnitude of Hg^0^ uptake fluxes over this time period; for example, Hg^0^ has decreased by ∼1–2% per year over North America and Europe from the 1990s to 2010s.^33^ To compare simulations directly with Hg fluxes, it would be necessary to conduct longer term simulations or reduce the dataset to a selected time period. Instead, we compare modeled Hg^0^ dry deposition velocities to calculated annual mean Hg^0^ dry deposition velocities 
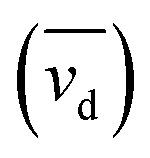
 from the observations, using:
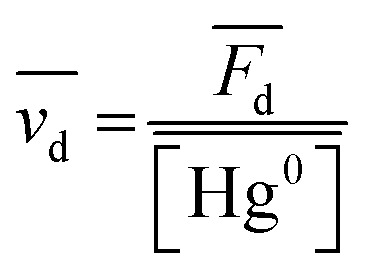



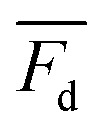
 is the measured litterfall or total foliar uptake flux and 
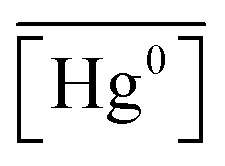
 is the annual mean atmospheric Hg^0^ concentration. The atmospheric Hg^0^ concentrations are taken directly from the measurement study, when reported. Otherwise, we searched for nearby atmospheric measurement sites for the relevant time period in the Atmospheric Mercury Network (AMNet),^34^ Canadian Air and Precipitation Monitoring Network (CAPMoN),^35^ European Monitoring and Evaluation Programme (EMEP),^36^ and Global Mercury Observation System (GMOS).^37^ Hg^0^ observations are generally reported in units of ng m^−3^ at standard temperature and pressure (STP, 273 K and 1 atm), whereas litterfall measurements would have units of μg per m^2^ per year based on local conditions. Therefore, we apply a temperature and pressure correction using MERRA-2 meteorological data^38^ to adjust annual mean measured Hg^0^ concentrations from STP to local conditions. The applied correction yields 3 to 39% decreases in Hg^0^ concentrations at local conditions compared to STP.

**Fig. 1:**
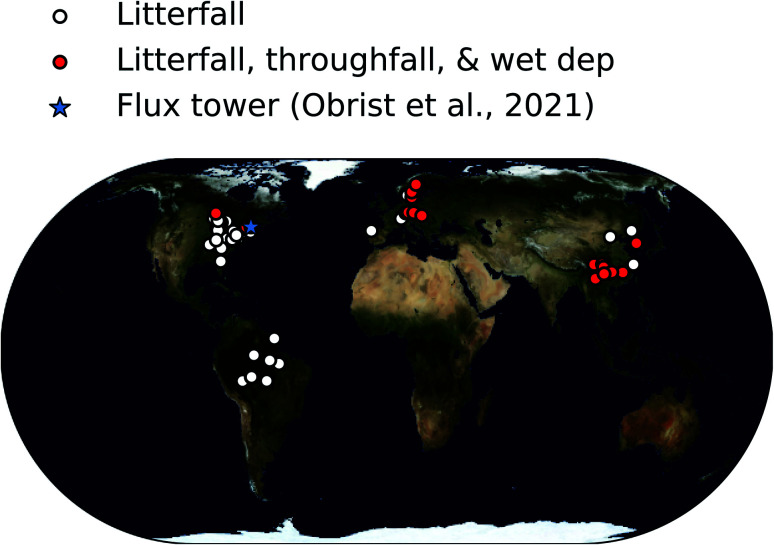
Map showing locations of forest sites where Hg^0^ vegetation uptake fluxes were measured in the compiled database.

In total, we determined dry deposition velocities at 92 sites based on litterfall measurements, at 33 sites based on total foliar uptake, and at one site based on micrometeorological measurements.^26^ The micrometeorological measurements were downloaded online^39^ and analyzed based on annual mean values of dry deposition fluxes and concentrations. The locations of these studies are shown in Fig. 1.

Several issues must be considered when comparing modelled Hg^0^ dry deposition velocities with the different measurement datasets (Table 1). Litterfall measurements do not account for atmospheric Hg^0^ that is translocated from foliage into woody tissues, as well as additional uptake from lichens, mosses, and soils.^6^ Therefore, we consider litterfall measurements to be a lower bound for total Hg^0^ dry deposition to a forest. Total foliar uptake measurements include an additional contribution from throughfall; however, throughfall fluxes include a fraction of previously dry deposited Hg^2+^ that is washed off the leaf surface, and thus may not be directly comparable with modelled Hg^0^ deposition. The contribution of Hg^2+^ dry deposition to throughfall likely varies spatially, with potentially higher contributions in areas close to anthropogenic Hg^2+^ emissions. One study in the Southeastern Tibetan Plateau region identified a 34–82% contribution from Hg^0^ to throughfall using isotopic evidence.^25^ Micrometeorological measurements of net ecosystem Hg^0^ exchange may be the most accurate proxy of total Hg^0^ vegetation uptake.^26^ Since net Hg^0^ exchange is measured, derived fluxes can include a negative contribution from soil Hg^0^ emissions; yet, Obrist *et al.*^26^ did not find any periods of net Hg^0^ emissions from the forest floor during their study. Given the uncertainties of the comparison between modelled Hg^0^ dry deposition and Hg vegetation uptake measurements, we use other available measurements (*e.g.*, of atmospheric Hg concentrations) as an additional independent constraint on Hg^0^ dry deposition (Sections 3.2 and 3.3).

The exchange of Hg between the canopy and atmosphere is bi-directional;^40^ all measurement methods studied here (litterfall, throughfall, and net ecosystem exchange) determine the net uptake flux rather than the gross uptake flux. The GEOS-Chem model does not explicitly include a parametrization for re-emission of Hg^0^ from foliar surfaces, so the modelled deposition fluxes should also be considered as net fluxes. GEOS-Chem calculates Hg^0^ emission fluxes from soil separately from the dry deposition scheme, using a parametrization depending on solar radiation and soil Hg concentration.^16^

### 2.2 GEOS-Chem description

We employ version 12.8.1 of the chemical transport model GEOS-Chem, whose Hg simulation is described by Horowitz *et al.*^2^ We run the mercury simulation globally at 2.0° × 2.5° resolution and 47 vertical layers. The model's atmospheric transport is driven by MERRA-2 assimilated meteorological data.^38^ The model calculates atmospheric mercury transport in three tracers: elemental mercury, Hg^0^, divalent mercury, Hg^2+^, and particulate-bound divalent mercury, Hg^P^. Most of the simulations in this study use the GEOS-Chem v12.8.1 Hg chemical scheme, which considers bromine (Br) to be the primary Hg^0^ oxidant^2^ and employs monthly mean Br oxidant concentrations from Schmidt *et al.*^41^ Additional simulations (see Section 2.4 and Table 2) were conducted in version 12.8.1 of GEOS-Chem but with the new Hg chemical mechanism from Shah *et al.*^3^ In the updated chemical mechanism, new reactions were added for the two-step oxidation of Hg^0^ to Hg^2+^ and the photolysis of gas phase Hg^+^ and Hg^2+^ compounds, based on new laboratory and computational data.^42,43^ Whereas Br was the primary Hg^0^ oxidant in the prior chemical scheme,^2^ Br and hydroxyl (OH) radicals play comparable roles in the net oxidation of Hg^0^ in the new chemical scheme.^3^ Shah *et al.*^3^ also use updated Br oxidant fields that include the effect of sea-salt aerosol debromination and correct issues with heterogeneous recycling of Br radicals.^44^ In both chemical mechanisms, aqueous reduction of Hg^2+^ to Hg^0^ is parametrized as a function of the NO_2_ photolysis rate and organic aerosol concentration, with a tuning parameter employed to optimize agreement with Hg^0^ observations.^2^ Partitioning between gaseous and particulate Hg^2+^ is calculated according to Amos *et al.*^45^ as a function of temperature and mass concentration of fine particulate matter (PM2.5). Wet deposition removes Hg^2+^ and Hg^P^ from the atmosphere according to gas^45^ and particulate^46^ parametrizations.

**Table 2:** Description of GEOS-Chem Hg simulations

Simulation name	Description	Hg^0^ biological reactivity (*f*_0_)	Reduction coefficient^a^ (*α*)
BASE	Reference GEOS-Chem version 12.8.1	10^−5^	0.16
OBRIST	Matching flux tower measurements at Harvard Forest^26^	3 × 10^−5^	0.16
OBRIST_R	Matching Obrist *et al.*^26^ measurements; increased Hg^2+^ reduction	3 × 10^−5^	0.33
AMAZON_L	Lower bound for Amazon: matching Hg litterfall flux from sites in Fostier *et al.*^31^	9 × 10^−5^ (Amazon)	0.33
		3 × 10^−5^ (elsewhere)	
AMAZON_U	Upper bound for Amazon: matching total foliar uptake of Hg from Fostier *et al.*^31,90^	0.2 (Amazon)	0.33
		3 × 10^−5^ (elsewhere)	
NEWCHEM	Reference GEOS-Chem simulation based on new Hg chemical scheme from Shah *et al.*^3^	10^−5^	0.004^b^
NEWCHEM_D	Increasing dry deposition to parameters from AMAZON_U; new chemistry^3^	0.2 (Amazon)	0.010^b^
		3 × 10^−5^ (elsewhere)	

a In GEOS-Chem, the photoreduction rate of aqueous-phase Hg^2+^–organic complexes is parametrized as *α j*_NO_2__ [OA] [Hg^2+^(aq)], where *α* is a tuned coefficient, *j*_NO_2__ is the local photolysis rate of NO_2_, and [OA] is the mass concentration of organic aerosol at STP.

b In the new chemical scheme, photoreduction of aqueous Hg^2+^–organic complexes is parametrized differently, as *α j*_NO_2__ [Hg^2+^P (org)], where [Hg^2+^P (org)] is the concentration of particulate Hg^2+^–organic complexes. Thus, the *α* coefficients cannot be directly compared with earlier GEOS-Chem versions.

The Hg^0^ dry deposition scheme in GEOS-Chem uses a resistance-based approach,^19,20^ similar to many other chemical transport models.^15,18^ Dry deposition is parametrized assuming three types of resistances in series: aerodynamic, boundary, and surface resistance. Aerodynamic and boundary resistances depend on grid scale meteorological variables (*e.g.*, temperature and windspeed), whereas surface resistance depends on the land use category, surface parameters (*e.g.*, leaf area index, LAI), chemical compound-specific parameters, and meteorology. The surface resistance is determined by calculating the effect of parallel resistances of vegetation stomata, cuticles, lower canopy surfaces, and the ground surface (including soil and leaf litter). To incorporate compound-specific effects, the Henry's law constant (H*) and biological reactivity (*f*_0_) of a compound are used as scaling factors in the resistance calculations (see Section S1† for full set of equations included in the dry deposition model). Compounds that are more soluble (higher H*) and/or more biologically reactive (higher *f*_0_) have faster dry deposition velocities,^20^ since dissolution and reaction are two parallel pathways for deposition to surfaces (Section S1†). A grid cell in GEOS-Chem can contain multiple land use categories; the dry deposition velocity is computed separately for each land use category within a grid cell and then averaged with area-weighting to produce the overall dry deposition velocity in a grid cell.

The implementation of dry deposition in GEOS-Chem uses 11 land use categories, with the categories relevant to this paper being deciduous forests, coniferous forests, and tropical rainforests.^47^ Since information about grid surface parameters in GEOS-Chem (*e.g.*, LAI) is grouped into 73 land use categories according to Gibbs,^48^ for the dry deposition scheme each of the 73 land categories is assigned to one of the 11 dry deposition categories. We use a reprocessed version of the Moderate Resolution Imaging Spectroradiometer (MODIS) LAI product^49^ in the dry deposition scheme. For Hg^0^ in GEOS-Chem, H* is set to 0.11 M atm^−1^ (ref. 50) and *f*_0_ was originally set to 10^−5^ to match the observations over North America available at the time.^51^ Over the ocean, Hg^0^ dry deposition is calculated as part of the air–sea gas exchange model^52^ instead of through the resistance-based scheme. Gaseous Hg^2+^ is biologically unreactive (*f*_0_ = 0) but highly soluble (H* = 10^14^ M atm^−1^) and the dry deposition of particulate Hg is calculated according to aerosol deposition scheme.^53,54^

Gridded emissions from anthropogenic sources are based on the 2015 inventory^55,56^ prepared for the 2018 Global Mercury Assessment.^1^ All other emissions follow Horowitz *et al.*^2^ Monthly mean surface ocean Hg concentrations are taken from Horowitz *et al.*,^2^ based on two-way coupling of GEOS-Chem with the MITgcm 3-D mercury ocean model.^57^ To account for prompt recycling of Hg^2+^, 20% of Hg^2+^ wet and dry deposition to terrestrial surfaces are re-emitted to the atmosphere as Hg^0^ directly after depositing.^51^

### 2.3 Offline dry deposition model

We ported the GEOS-Chem dry deposition code to Python so that it could be run with meteorological inputs in offline calculations (https://doi.org/10.5281/zenodo.6498126). This offline model enables quicker simulations of dry deposition velocities than running the full online chemistry-transport model. The inputs to the model are hourly MERRA2 meteorological data for 2015 (including temperature, wind speed, cloud fraction, radiation fluxes), land surface information for the 73 land categories,^48^ and weekly-averaged satellite-derived LAI maps for 2015.^49^ The output of the offline model is hourly dry deposition velocity maps for 2015. With an hourly time resolution for meteorological inputs, the offline model shows small enough errors for the current study application compared to online GEOS-Chem velocities (mean grid cell error ∼ 0.1%) (ESI,† Section S2).

In this study, we run offline simulations with the biological reactivity (*f*_0_) of Hg^0^ varying from 10^−5^ to 1 and compare the resultant deposition velocity maps with the observational database. We focus on uncertainties in *f*_0_ since this parameter covers multiple potential biological reaction processes in vegetation uptake and therefore would be difficult to determine experimentally. In GEOS-Chem, *f*_0_ of Hg^0^ has been set to 10^−5^ based on Selin *et al.*,^51^ yet other modelling exercises use a higher Hg^0^ biological reactivity of 0.1 and 0.2.^58,59^ We chose to vary the chemical compound-specific parameter *f*_0_ instead of general resistance parameters within the dry deposition scheme, since the resistance parameters have been validated in model comparisons with other chemical compounds^60^ and would require a wider scope of chemical compounds measurements to evaluate (see Section S5† for further discussion).

The compiled observations are made at forest sites, whereas the corresponding model grid cells (2.0° × 2.5°) can cover multiple land types. We account for the specific land use category of the observation site in the offline model calculations following an approach by Silva and Heald.^60^ To do so, we categorize each observation site within the 11 land type categories used in the dry deposition scheme (*e.g.*, broadleaf, coniferous, and tropical rain forest). We alter the offline model's inputs so that the land type at the observation site accounts for 100% of the model grid cell. The LAI of the grid cell is set to the average LAI of the observed forest category within the grid cell. The surface roughness of the grid cell is adjusted to 1 m when it drops below that threshold, since 1 m is representative of forested areas.^61^ We present the effects of the land-surface adjustment in the ESI (Section S3†). In the case of Hg^0^, this adjustment is especially important for coastal grid cells, where the low deposition velocity over the water-covered sub-grid cell reduces the overall grid cell deposition velocity.

### 2.4 GEOS-Chem simulations

We conducted a four-year spinup of the GEOS-Chem Hg model (2010–2013) to create initial conditions for the subsequent simulations. Based on the comparison between the offline dry deposition model and observations (presented in Section 3.1), we ran two-year online simulations (2014–2015) for the different dry deposition settings (Table 2). Allowing for a one-year equilibration in the troposphere, we analyze year 2015 in the simulations. A reference BASE simulation (*f*_0_ = 10^−5^) is conducted with GEOS-Chem version 12.8.1. Three more simulations with different *f*_0_ parameter values were run: an OBRIST simulation with *f*_0_ set to 3 × 10^−5^ globally, as well as simulations where *f*_0_ is increased regionally in the Amazon rainforest to 9 × 10^−5^ (AMAZON_L simulation) and 0.2 (AMAZON_U simulation) and set to 3 × 10^−5^ elsewhere.

In order to balance the enhanced removal of Hg^0^ due to dry deposition in the OBRIST simulation, we adjust the reduction rate of Hg^2+^ to match annual mean observations of atmospheric Hg^0^ concentrations in the Northern Hemisphere in the OBRIST_R simulation. The larger reduction rate coefficient is also used in the AMAZON_L and AMAZON_U simulations. The Hg^2+^ reduction rate coefficient has been used as a tuning parameter in past developments of the GEOS-Chem model.^2,3^ Furthermore, here we have applied an offline three-box atmospheric model to reduce the computational expense of tuning the reduction rate (Section S4†).

To compare the effects of increased Hg^0^ dry deposition in the updated Hg chemistry mechanism,^3^ we run a reference simulation for the updated chemistry (NEWCHEM), as well as a simulation accounting for higher Hg^0^ dry deposition globally (*f*_0_ = 3 × 10^−5^) and in the Amazon (*f*_0_ = 0.2) and faster aqueous Hg^2+^ reduction (NEWCHEM_D) (Table 2). All emission settings remain unchanged from earlier simulations (Section 2.2).

As an illustrative scenario, we investigate the impact of the Amazon transitioning from rainforest to savannah (*savannization*) on the global vegetation uptake of Hg and the atmospheric Hg budget in GEOS-Chem. We follow the approach used by Alves de Oliveira *et al.*^62^ as a worst-case scenario for Amazon savannization due to deforestation and climate change. To set up the simulation, we replace all tropical rain forest land cover in South America with savanna land cover. We substitute Amazon LAI with mean time-varying LAI from current savanna areas in South America. Since the Hg budget of the savannization simulation is compared to AMAZON_U, *f*_0_ is set to 3 × 10^−5^ (no rainforest is present in Amazon, so the regional *f*_0_ setting is not applied).

## 3. Results and discussion

### 3.1 Comparison of offline model with vegetation uptake measurements

**Fig. 2:**
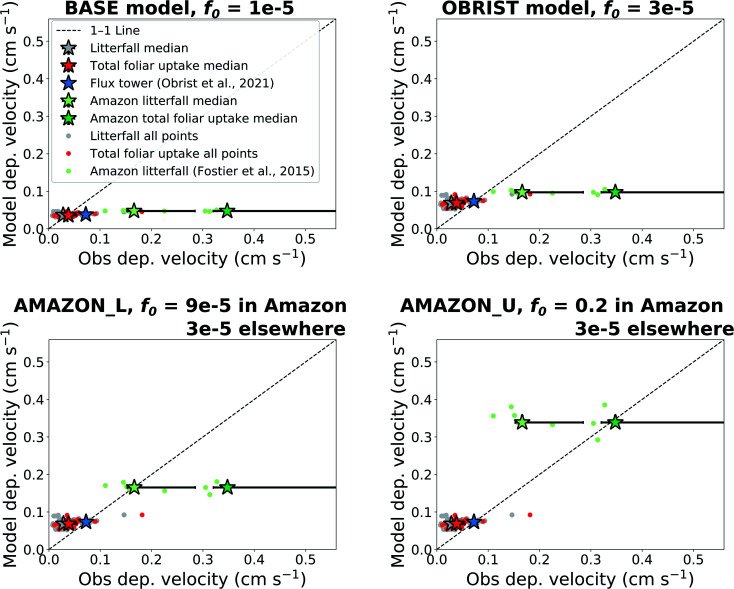
Comparing observed dry deposition velocities with modelled values extracted from the location coordinates. Four offline model settings are presented, with varying assumptions for the biological reactivity (*f*_0_) of Hg^0^ in the dry deposition scheme. Individual site measurements are indicated with filled circles and overall medians of measurement types are indicated with filled stars. Error bars show the interquartile range of measurements over different measurement locations. Model interquartile ranges are generally smaller than the size of the markers.

We compare the dry deposition velocities predicted by the offline model simulations to observations of litterfall (separating outside and inside the Amazon), total foliar uptake, and flux tower measurements (Fig. 2). The interquartile range of Hg^0^ dry deposition velocities predicted by the BASE version of GEOS-Chem (*f*_0_ = 10^−5^), 0.035–0.039 cm s^−1^, matches well with litterfall measurements outside the Amazon, 0.020–0.041 cm s^−1^ (Fig. 2). These measurements are located in mainly temperate and boreal forests in North America, Europe, and East Asia. Previous studies have also found good agreement between GEOS-Chem Hg^0^ dry deposition and a USA subset of litterfall measurements.^9,16^ When the throughfall dry deposition component is added to litterfall, the median dry deposition velocity of observations increases by ∼36%, from 0.028 cm s^−1^ to 0.038 cm s^−1^. The interquartile ranges of BASE (0.034–0.041 cm s^−1^) also overlap with total foliar uptake observations (0.030–0.055 cm s^−1^). The modelled dry deposition velocity at Harvard Forest (0.038 cm s^−1^) underpredicts the flux tower measured value^26^ (0.072 cm s^−1^) by a factor of 1.9. Even larger underestimates are seen when comparing the BASE model to deposition velocities derived from Amazon litterfall (−72% from observation median) and Amazon total foliar uptake (−87% from observation median).^31^

When the value of *f*_0_ is adjusted to 3 × 10^−5^ (OBRIST simulation), the model matches the Hg^0^ deposition velocity derived from the flux tower measurements. In this case, the model overestimates median total foliar uptake deposition velocities by 78%. This model-based estimate is only based on one flux tower measurement site, and more flux tower measurements of Hg^0^ exchange over forests would be necessary to confirm the stronger sink measured by Obrist *et al.*^26^ Nevertheless, the larger dry deposition velocities measured by flux tower measurements compared to litterfall could be explained by litterfall measurements not accounting for uptake from woody tissues, lichens and mosses.^6^

The model continues to underestimate Amazon litterfall measurements when *f*_0_ of Hg^0^ is set globally to 3 × 10^−5^ (OBRIST) (Fig. 2). A previous study with a different atmospheric Hg model that uses a resistance-based approach to dry deposition (GEM-MACH-Hg) also underestimated deposition fluxes inferred from tropical rainforest litterfall measurements.^5^ We therefore applied a new regional *f*_0_ parameter for the rainforest land type in South America. Setting *f*_0_ to 9 × 10^−5^ within only the Amazon rainforest and retaining *f*_0_ = 3 × 10^−5^ elsewhere (AMAZON_L simulation), leads to a match for the Amazon litterfall deposition velocity (0.17 cm s^−1^). We take this value as a lower bound estimate for Hg^0^ dry deposition in the Amazon, since litterfall does not account for all vegetation uptake (Table 1). As an upper bound estimate for the Amazon dry deposition velocity, we use the total foliar uptake estimate, litterfall + throughfall − open field wet deposition (49 + 72 − 18 = 103 μg per m^2^ per year), reported by Fostier *et al.*^31^ Dividing this by the atmospheric Hg^0^ concentration measured in the GMOS station at Manaus, we calculate a deposition velocity of 0.35 cm s^−1^. This estimate is considered an upper bound for Amazon Hg^0^ uptake since lower throughfall fluxes (13.7–23.6 μg per m^2^ per year) have been reported for other regions of the Brazilian Amazon.^63^ A recent study from the Peruvian Amazon measured a slightly higher total foliar uptake flux (128 μg per m^2^ per year); however, the study area is impacted by regional artisanal and small-scale gold mining (ASGM) emissions.^64^ The upper observed bound of Amazon dry deposition velocity from Fostier *et al.*^31^ would correspond to a *f*_0_ value of 0.2 (AMAZON_U simulation).

Instead of altering *f*_0_, another approach to adjusting the Hg^0^ dry deposition velocity for the Amazon rainforest would have been to alter the dry deposition canopy resistances for the rainforest land category. We show that an equivalent solution for Hg^0^ deposition in the Amazon can be found by adjusting resistances (Section S5†). However, since adjusting resistances would affect all dry depositing compounds in GEOS-Chem and perturb their atmospheric budgets, for this study we proceeded with adjustments to the Hg^0^-specific parameter, *f*_0_. Given that both approaches would yield the same Hg^0^ dry deposition velocities, the choice of approach should not affect the subsequent GEOS-Chem analyses in this study. Nevertheless, this issue should be revisited with a more extensive comparison of multiple chemical compounds, as GEOS-Chem (Section S5†) and another model with a resistance-based approach^65^ have also been found to underestimate the ozone dry deposition velocity in the Amazon.

### 3.2 Seasonality of atmospheric Hg^0^

**Fig. 3:**
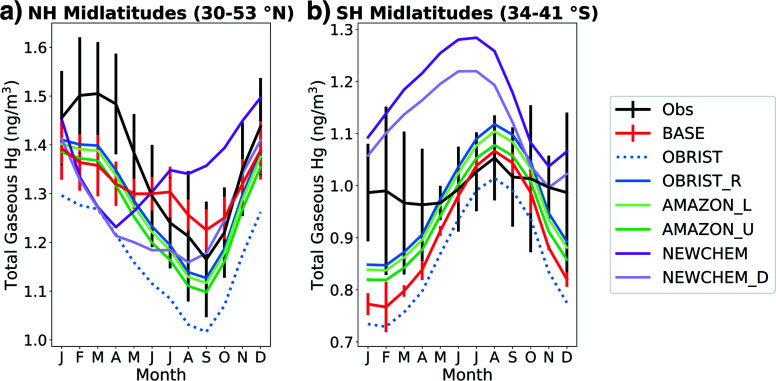
Seasonal cycle of surface total gaseous mercury (TGM) concentrations in the (a) Northern Hemisphere (NH) midlatitudes (16 observation stations) and (b) Southern Hemisphere (SH) midlatitudes (3 observation stations). Lines indicate monthly means of simulated or observed Hg^0^ at station locations and error bars indicate standard deviations between measurement sites. To improve plot clarity, model error bars are only shown for the BASE simulation. Measured atmospheric Hg concentrations are sourced from compilations^18,91^ and are courtesy of Hélène Angot.

We compare the atmospheric Hg^0^ seasonal cycle from online GEOS-Chem simulations (Table 2) with observations in Fig. 3. Under the BASE simulation, GEOS-Chem is within the 1*σ* spatial variability of Northern Hemisphere (NH) midlatitude observations in all months except April (Fig. 3a). However, the seasonal amplitude of the BASE model (0.17 ng m^−3^) is only half the observed seasonal amplitude (0.34 ng m^−3^), showing a weaker summertime minimum in total gaseous mercury (TGM). In the BASE version of GEOS-Chem, seasonality in Hg oxidation drives the summertime minimum in the NH.^2,66^ In the OBRIST simulation, with increased Hg^0^ dry deposition, the model's seasonal amplitude (0.28 ng m^−3^) is closer to observations (0.34 ng m^−3^) although the mean concentrations are biased low compared to observations. This bias can be offset by adjusting the reduction of Hg^2+^ to Hg^0^ in organic aerosol, which has been done to tune previous GEOS-Chem Hg model versions.^2,3^ The OBRIST_R simulation illustrates that when the Hg^2+^ reduction rate coefficient is increased by a factor of 2.1, the model based on flux tower measurements can match both the mean (within 1*σ*) and the seasonal amplitude of observed Hg^0^ in the NH midlatitudes (Fig. 3a). More information about the tuning procedure for Hg reduction, as well as comparisons with observed Hg^0^ concentrations globally, can be found in the ESI† (Section S4 and Fig. S9†).

The improved agreement of the modelled seasonal cycle upon increasing the strength of Hg^0^ dry deposition supports previous work suggesting that the Hg^0^ seasonal cycle in the NH is driven mainly by the vegetation sink, not oxidation chemistry.^4,5^ The simulations that further increase Amazon uptake of Hg^0^ (AMAZON_L and AMAZON_U) are similar in seasonality to the OBRIST_R simulation in the NH midlatitudes. Thus, the three simulations OBRIST_R, AMAZON_L, and AMAZON_U can all be compatible with NH midlatitude Hg^0^ observations and suggest that the vegetation uptake in the BASE model is too small.

Shah *et al.*^3^ updated the Hg chemical scheme in GEOS-Chem (NEWCHEM), leading to substantial differences in the seasonal cycle of TGM in the NH midlatitudes (Fig. 3a). In the NEWCHEM simulation, the minimum Hg^0^ concentrations occur in April, whereas BASE chemistry leads to a minimum in September. This difference is caused by the seasonality of Br oxidant concentrations; NEWCHEM uses updated Br fields^44^ that show maximum concentrations in the NH in late winter–spring, whereas the Br fields used by BASE^41^ are maximum in summer. When dry deposition is enhanced in the new chemistry version (NEWCHEM_D), the minimum TGM concentration is shifted to August, closer to observations. The seasonal amplitude of NEWCHEM_D (0.25 ng m^−3^) is similar to the updated simulations using the previous chemical mechanism (OBRIST_R, AMAZON_L, and AMAZON_U), illustrating that NH midlatitude TGM seasonality can be a useful constraint for Hg^0^ dry deposition.^4^ However, NEWCHEM_D still shows too low TGM concentrations throughout the boreal spring (March–May), suggesting that further investigation into the uncertainties of atmospheric Hg chemistry is required.

In the Southern Hemisphere (SH) midlatitudes, the observations show a relatively flat seasonal cycle in Hg^0^ (amplitude of 0.09 ng m^−3^) (Fig. 3b). Jiskra *et al.*^4^ have attributed this lack of seasonality to less land area in the SH, and thus a more minor vegetation sink. In contrast, the BASE version of GEOS-Chem shows a strong seasonal cycle (amplitude of 0.30 ng m^−3^), with a wintertime maximum and summertime minimum likely driven by oxidation chemistry.^2,3^ The seasonal amplitude is largely unchanged (0.29 ng m^−3^) when dry deposition is increased in the OBRIST simulation, because of the limited influence of land in the SH. When the reduction of Hg^2+^ is increased in OBRIST_R, the seasonality is slightly flattened, with an amplitude of 0.27 ng m^−3^. Further adjustments to the Amazon dry deposition flux (AMAZON_L and AMAZON_U) lead to reductions in mean SH midlatitude TGM, but limited changes in seasonal amplitude. Simulations with the new chemical mechanism (NEWCHEM and NEWCHEM_D) also show too strong TGM seasonality in the SH (amplitudes of 0.22 and 0.25 ng m^−3^) and generally overestimate SH midlatitude concentrations (annual mean bias of +0.18 and +0.13 ng m^−3^). Uncertainties with the GEOS-Chem Hg chemical scheme as well as seasonality of Br oxidant concentrations could be responsible for the bias in TGM seasonality in the SH.^3^ A further uncertainty is the seasonality of Hg^0^ emissions from the ocean, which would likely be a dominating source in the SH.^2^

### 3.3 Atmospheric Hg^0^ in South America

**Fig. 4:**
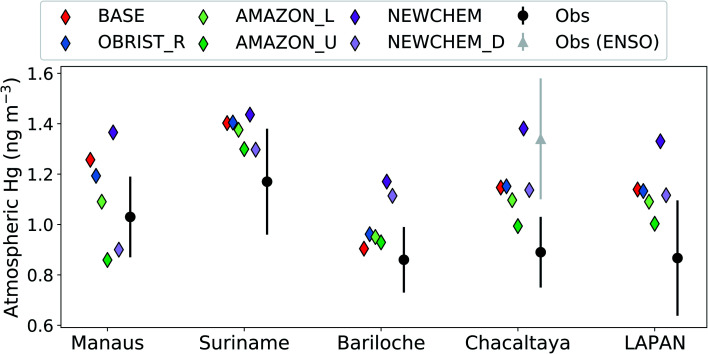
Comparing GEOS-Chem simulations to available South American observations of atmospheric Hg (LAPAN stands for Latin American Passive Sampling Network). Total gaseous mercury (TGM) concentrations for simulations and observations are compared at each site, except Suriname for which gaseous Hg^0^ (GEM) concentrations are compared. Error bars for observations indicate the 1*σ* temporal variability, except for LAPAN which shows the 1*σ* spatial variability between 22 sites.

Previous validations of GEOS-Chem^2,3,66^ have paid limited attention to South America, where until now very few measurements were available. Given the importance of the Amazon region as an area of dry deposition (accounting for 29% of the global Hg^0^ dry deposition to land in AMAZON_U), we evaluate GEOS-Chem results against recent atmospheric Hg measurements from South America^37,67,68^ (Fig. 4). Atmospheric Hg^0^ observations from Chacaltaya, a mountain-top observatory in the Bolivian Andes, showed an increasing trend between normal (July 2014–May 2015) and El-Niño Southern Oscillation (ENSO) conditions (June 2015–February 2016).^67^ We focus our comparison on normal conditions at Chacaltaya, since the Hg^0^ ocean emissions in GEOS-Chem would not be representative of ENSO conditions.^2^ In general, Hg concentrations measured in stations surrounded by tropical rainforest (Manaus, Suriname, and Chacaltaya) are all overestimated by the BASE version of GEOS-Chem (BASE mean over stations: 1.27 ng m^−3^*vs.* observed mean: 1.03 ng m^−3^). Only at the midlatitude site Bariloche, which would be less affected by the Amazon vegetation uptake sink, is the BASE model within the measurement 1*σ* variability. Passive Hg samplers, as part of the Latin American Passive Air sampling Network (LAPAN) network, are a new source of information for Hg cycling in South America.^68^ We compare the mean of passive-sampler measured annual Hg concentrations from 22 background locations in South America with model simulations (Fig. 4). The BASE simulation also overestimates the mean Hg concentration from the passive sampler network (1.14 ng m^−3^*vs.* 0.87 ng m^−3^), supporting the comparison with other measurement stations.

The OBRIST_R simulation with increased Hg^0^ biological reactivity globally remains outside the measurement 1*σ* variability at South American sites, except for the midlatitude site Bariloche. With the adjustment to Amazon Hg^0^ biological reactivity in AMAZON_L and AMAZON_U, the bias in GEOS-Chem predictions are generally reduced at the South American sites (Fig. 4). Overall, the AMAZON_U simulation seems to agree best with Hg^0^ measurements, lying within the 1*σ* variability at all measurement sites except for Manaus, where the AMAZON_L simulation is closest. Our comparison with observations of atmospheric Hg^0^ concentrations in South America supports the conclusion that the vegetation uptake of Hg in the Amazon is currently underestimated in GEOS-Chem. In addition to dry deposition uncertainties, atmospheric Hg concentrations over South America may also be affected by uncertainties in soil re-emissions, artisanal and small-scale gold mining emissions, air–sea exchange, and volcanic emissions.^1,15,67^ Nevertheless, correcting GEOS-Chem to be in line with Amazon litterfall and throughfall data, helps reduce the South American Hg^0^ bias in GEOS-Chem from +0.21 ng m^−3^ (BASE) to +0.05 ng m^−3^ (AMAZON_U), averaged over the observations in Fig. 4. The new Hg chemical scheme,^3^ generally leads to higher Hg concentrations in the SH (Fig. 3b and 4). However, as with the old chemical scheme,^2^ we find that the enhancement of dry deposition globally and over the Amazon improves the overall South American Hg^0^ bias from +0.37 ng m^−3^ (NEWCHEM) to +0.15 ng m^−3^ (NEWCHEM_D).

### 3.4 Wet deposition fluxes of Hg

An additional observational constraint on atmospheric Hg cycling is measured wet deposition fluxes. Due to the relative insolubility of Hg^0^, wet deposition fluxes depend on the atmospheric concentrations of Hg^2+^. Our adjustments to increase the Hg^0^ dry deposition to land consequently result in less available Hg^0^ for oxidation and thus decrease the wet removal sink of Hg^2+^. The BASE version of GEOS-Chem underestimates observed annual mean wet deposition globally (7.5 μg m^−2^*vs.* 8.3 μg m^−2^, Fig. S10†). The mean simulated wet deposition decreases in AMAZON_U (4.5 μg m^−2^), increasing the model bias. However, other processes may explain the deviation between GEOS-Chem and wet deposition measurements. For example, GEOS-Chem underestimates Hg^2+^ in the free troposphere, likely due to uncertainties in atmospheric Hg oxidation, leading to less Hg removal by convective storms.^3,69^ Indeed, when the new chemistry scheme is applied from Shah *et al.*^3^ (NEWCHEM_D), Hg wet deposition increases over Europe compared to AMAZON_U (4.8 μg m^−2^*vs.* 3.4 μg m^−2^, Fig. S12†), but remains too low globally (4.9 μg m^−2^, Fig. S10†). Other studies have cited the difficulty of resolving Hg^2+^ hotspots and transport in global models,^70,71^ the speciation of anthropogenic Hg emissions,^33,72^ and the lack of Hg in coarse particles in GEOS-Chem^73^ as possible reasons for the wet deposition underestimation. Further analysis and observational constraints are required to investigate the influence of these uncertainties on Hg wet deposition.

### 3.5 Importance of Amazon rainforest as a Hg^0^ sink

Both litterfall flux and atmospheric Hg^0^ concentration measurements suggest that the Amazon rainforest is currently an important net Hg^0^ sink, yet this sink is at risk due to continued deforestation and climate change.^14,74^ Two tipping points have been suggested that could cause the rainforest to transition into a savannah biome: a temperature increase of 4 °C above preindustrial or 20–40% deforestation in the region.^75^ Once these thresholds are crossed, regional moisture recycling weakens to the extent that there is not enough available water to support tropical rainforest biomes.

**Fig. 5:**
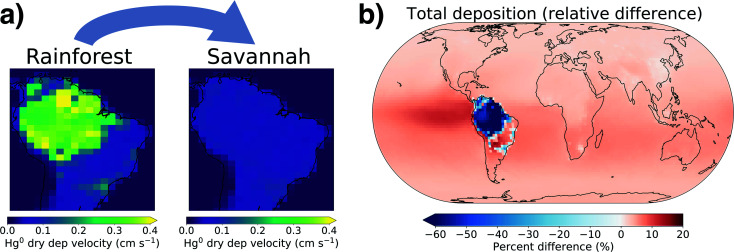
(a) GEOS-Chem modeled Hg^0^ dry deposition velocities shown for the Amazon under current land cover (left) and total conversion to savannah (right). (b) Map of the simulated change in total Hg deposition flux due to savannization.

To investigate the effect of Amazon savannization on regional and global Hg cycling, we simulated an extreme scenario where the entire Amazon rainforest is converted to savannah land cover, following the approach of Alves de Oliveira *et al.*^62^ In the AMAZON_U simulation, median modelled Hg^0^ dry deposition velocities in the Amazon rainforest are 0.3 cm s^−1^, while in the savannah scenario this decreases to 0.07 cm s^−1^ (Fig. 5a). Correspondingly, the Hg^0^ dry deposition over the Amazon region decreases by 63% in the savannah scenario (Fig. 5b), from 653 Mg per year to 246 Mg per year. Total Hg deposition to the ocean increases by 283 Mg per year, mostly spread evenly across the SH but with an intensified band in the eastern equatorial Pacific. In the absence of the Amazon rainforest uptake sink, the total Hg burden in the atmosphere increases by 194 Mg (+5%) compared to current conditions.

In South America, the major direct anthropogenic source of Hg is from ASGM, emitting 340 Mg per year to the atmosphere.^1^ Much of the ASGM activities occur within the Amazon region.^76,77^ Due to uptake of Hg by vegetation, terrestrial ecosystems close to ASGM sources are threatened by high Hg exposures.^64^ The ASGM Hg^0^ emissions may change in the future as parties to the Minamata Convention on Mercury are obligated to take steps to reduce Hg use in ASGM.^78^ To check how much of the rainforest Hg^0^ sink is caused by regional emissions, we ran two scenarios with no South American anthropogenic Hg emissions and either existing rainforest conditions or full savannah conversion. Even when regional emissions are removed, savannization results in an additional 238 Mg per year Hg transferred to the ocean (83% of the effect calculated with current South American emissions). The Amazon rainforest is thus not only a sink of regional Hg but also global background Hg, due to the long atmospheric lifetime of Hg^0^.

Previous work has identified several processes that mobilize Hg during Amazon deforestation.^31^ Soil erosion and mobilization increases in deforested areas, with annual Hg mobilization expected to increase by 20–25% between 2014 and 2030 in a Peruvian watershed affected by deforestation.^79^ Fires used to clear forested areas for agriculture have been estimated to emit 6.7 Mg Hg per year between 2000 and 2008 in the Brazilian Amazon.^80^ Deforested areas also show higher soil Hg emissions than forested areas, with soils releasing an estimated 2.3 g per Hg per ha in the first year after the fire.^7^ When scaled by the estimated deforestation rate from the Brazilian Amazon in 2021 (1.3 × 10^6^ ha per year),^81^ the additional soil source after deforestation would correspond to 3.0 Mg Hg per year. With our current study, we quantified another process leading to increased mobilization of Hg to marine ecosystems: a decreased land sink that leads to additional Hg deposition to the ocean. The estimated increased Hg deposition flux to the ocean (283 Mg per year) represents two-thirds of the total anthropogenic South American emissions (340 Mg per year), emphasizing the importance of Amazon rainforest trends for future Hg contamination. This shift in Hg deposition from land to the ocean increases the overall mobility of Hg in the surface environment since Hg in soil reservoirs has a longer mean residence time than in the surface ocean.^82^ Increased exposure to Hg health risks may not be limited to marine ecosystems, since deforestation can increase Hg methylation rates in freshwater aquatic ecosystems.^83^ Efforts to mitigate climate change and avoid deforestation will be crucial to avoid additional mobilization of Hg in the environment, meaning that the goals of the Minamata Convention intersect with other international treaties such as the Paris Climate Accords.^78^

## 4. Environmental and modelling implications

**Fig. 6:**
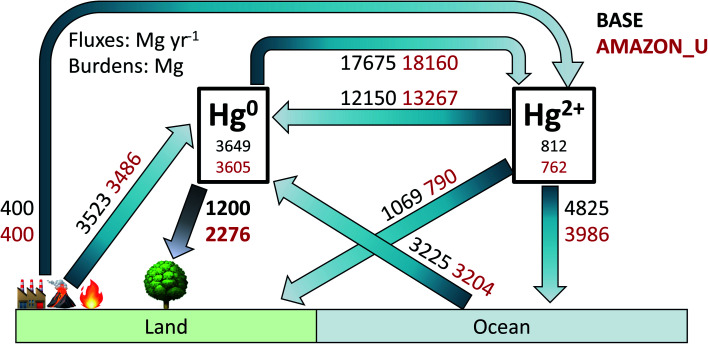
Atmospheric Hg budget for BASE and AMAZON_U simulations for year 2015. Atmospheric burdens are listed in units Mg and fluxes in Mg per year. The Hg^2+^ burden and fluxes include both gaseous and particulate Hg^2+^.

Fig. 6 illustrates the global atmospheric Hg budget under the original GEOS-Chem simulation BASE and the updated simulation AMAZON_U (other simulation budgets are shown in Table S2†). The atmospheric Hg^0^ burden (∼3600 Mg) remains similar in both simulations, given that Hg^2+^ reduction was increased to compensate for increased Hg^0^ deposition in AMAZON_U. Overall, dry deposition of Hg^0^ to land approximately doubles from 1200 Mg per year in BASE to 2276 Mg per year in AMAZON_U. The new global dry deposition flux to land (2276 Mg per year) lies in between the estimate from a previous global litterfall assessment (1180 Mg per year)^27^ and the estimate extrapolated globally from one flux tower measurement site over a midlatitude forest (3490 Mg per year).^26^ The new estimate from GEOS-Chem is within the lower end of the range (2705 ± 504 Mg per year) derived by a new assessment that considered uptake to moss, lichens, and woody tissues in addition to litterfall.^6^ Total Hg deposition to land in AMAZON_U (2960 Mg per year) also matches with the simulated value from GEM-MACH-Hg (2800 Mg per year).^5^ With increased vegetation uptake of Hg^0^, AMAZON_U shows improved seasonality of atmospheric Hg^0^ at NH midlatitude sites and reduced biases in Hg^0^ over South America, compared to the BASE simulation. In AMAZON_U, the increased flux of Hg^0^ dry deposition and reduced fluxes of Hg^2+^ deposition (−1118 Mg per year, −19% compared to BASE) helps resolve part of the model bias identified in isotopic Δ^200^Hg mass balance studies.^29,84^ The enhanced role of the land sink in the improved version of GEOS-Chem highlights the importance of considering biosphere-atmosphere feedbacks during climate change in future projections of Hg pollution. Our study also emphasizes the necessity of expanding litterfall monitoring as a lower bound estimate for the magnitude Hg^0^ uptake and conducting additional flux tower studies in different biomes to monitor net ecosystem exchange. Specifically, there are no litterfall flux measurements of Hg from rainforests in Africa or Southeast Asia, which would be invaluable to investigate whether similar uptake processes occur there as in the Amazon.

Our study has improved the Hg^0^ dry deposition parametrization by evaluating GEOS-Chem with a wider array of field evidence (79 studies) than the original parametrization.^51^ Nevertheless, the model is still not able to predict observed deposition velocities from individual studies and observed values generally show more variance than modelled values (Fig. 2 and Section S7†). This result indicates that important controls on vegetation uptake could be missing from the GEOS-Chem dry deposition parametrization. For example, the current parametrization^19,20^ does not include tree species type or moisture availability, which have been found to influence the variability of foliar Hg uptake across Europe.^10^ Other studies highlighted the importance of water availability on plant uptake of ozone, especially when considering the impacts of future climate change, and their expanded parametrizations may serve as examples for future GEOS-Chem development.^65,85^ GEOS-Chem does not explicitly treat the re-emission of Hg^0^ from foliage, which isotopic evidence suggests is around 30% of the gross uptake flux.^86^ Other studies have developed bi-directional models of Hg^0^ exchange in the canopy;^40,87^ however, the modelled seasonality of Hg^0^ re-emissions^88^ disagrees with recent net ecosystem exchange measurements from Harvard Forest.^26^ Further combined analysis of field measurements and model parametrizations is essential to implement improved atmosphere–biosphere exchange schemes in chemistry transport models.^15,89^

## 5. Conclusion

Dry deposition of Hg^0^ is a major removal pathway of atmospheric Hg and controls the influx of Hg to terrestrial ecosystems. We conducted model simulations in GEOS-Chem that correspond to different observational constraints of Hg^0^ vegetation uptake. The original BASE simulation agrees with litterfall and total foliar uptake data from outside of the Amazon, yet it cannot capture the seasonality of atmospheric Hg^0^ in the NH midlatitudes and it overestimates atmospheric Hg^0^ in South America. We suggest a higher biological reactivity of Hg^0^ (*f*_0_) than the BASE simulation: 0.2 within the Amazon rainforest, in line with available Amazon litterfall and throughfall data,^31^ and 3 × 10^−5^ elsewhere, in line with the existing flux tower study.^26^ This simulation (AMAZON_U) better matches the seasonality of NH midlatitudes Hg^0^ and agrees best with available atmospheric Hg^0^ observations in South America. The revised simulation leads to a global Hg^0^ land sink (2276 Mg per year) that is almost double the original simulation (1200 Mg per year). The Amazon rainforest contributes approximately 29% of the total Hg^0^ land sink worldwide, but continuing deforestation endangers this sink. In the extreme scenario where the entire rainforest is converted to savannah, an additional 283 Mg per year Hg is transferred to the ocean where it can bioaccumulate in the marine food chain. The improved version of GEOS-Chem can be applied in future studies to understand Hg cycle feedbacks between the terrestrial biosphere and the atmosphere.

## Conflicts of interest

There are no conflicts to declare.

## Supplementary Material

EM-024-D2EM00032F-s001

EM-024-D2EM00032F-s002
