# Supplementary material for: Evaluating atmospheric mercury (Hg) uptake by vegetation in a chemistry-transport model
Source: Environ Sci Process Impacts. 2022 Apr 22;24(9):1303–18. doi: 10.1039/d2em00032f (PMC9491292; doi:10.1039/d2em00032f)
Supplement: EM-024-D2EM00032F-s002 [file EM-024-D2EM00032F-s002.pdf]

Electronic Supplementary Material (ESI) for Environmental Science: Processes & Impacts.
This journal is © The Royal Society of Chemistry 2022

Supplementary Information

Evaluating atmospheric mercury (Hg) uptake by vegetation in a chemistry-transport model

Aryeh Feinberg*, Thandolwethu Dlamini, Martin Jiskra, Viral Shah, and Noelle E. Selin*
*Correspondence to: arifein@mit.edu (A.F.); selin@mit.edu (N.E.S.)

S1. Formulation of dry deposition model used in GEOS-Chem

The dry deposition model is a resistance-based empirical model, originally presented by Wesely (1989) and implemented in GEOS-Chem by Wang et al. (1998). In this section, we present the key equations relevant to the Hg^0 dry deposition scheme.

The overall deposition velocity in a grid cell (V_d) is calculated as the average of the dry deposition velocity of each land type (i) present in the grid cell (V_d^i), weighted by area fraction (A^i):

$$V_d = \sum_{i=1}^{73} A^i V_d^i \qquad (1)$$

The dry deposition velocities are calculated in a resistance-based approach, considering three types of resistance in series, the aerodynamic resistance (R_A), the sublayer resistance (R_B), and the bulk surface resistance (R_C^i):

$$V_d^i = \frac{1}{R_A + R_B + R_C^i} \qquad (2)$$

The aerodynamic resistance depends on meteorological conditions and does not vary between gas-phase compounds. The sublayer resistance also depends on meteorological conditions, as well as the diffusivity of the gas-phase compound. The algorithm to calculate the aerodynamic and sublayer resistances from grid scale meteorological variables can be found in the model code (https://github.com/arifein/offline-drydep). The bulk surface resistance is calculated as a function of in-series and parallel resistors:

$$\frac{1}{R_C^i} = \frac{1}{r_{ix}^i} + \frac{1}{r_{lu}^i} + \frac{1}{r_{dc}^i + r_{cl}^i} + \frac{1}{r_{ac}^i + r_{gs}^i} \qquad (3)$$

where r_{ix}^i is the internal stomatal resistance, r_{lu}^i is the cuticular resistance, r_{dc}^i is the aerodynamic resistance in the lower part of the canopy, r_{cl}^i is the lower canopy surface resistance (e.g., leaves, branches, and bark), r_{ac}^i is the resistance due to canopy height and density, and r_{gs}^i is the resistance at the ground surface (e.g., soil and leaf litter).

The internal stomatal resistance is calculated as:

$$r_{ix}^{i} = r_{ix}^{i'} \times a(T) \times b(\mathrm{LAI}^{i}, \theta, C_F) \frac{D_{\mathrm{H_2O}}}{D_{\mathrm{Hg^0}}} + \frac{1}{\frac{H_{\mathrm{Hg^0}}^{*}}{3000} + 100 f_0^{\mathrm{Hg^0}}} \qquad (4)$$

where $r_{ix}^{i'}$ is the minimum stomatal resistance for water vapour, $a(T)$ is a function depending on temperature, $b(\mathrm{LAI}^{i}, \theta, C_F)$ is a function depending on leaf area index (LAI), the solar zenith angle, and cloud fraction, D is the molecular diffusivity, $H_{\mathrm{Hg^0}}^{*}$ is the Henry's law constant of Hg^0, and $f_0^{\mathrm{Hg^0}}$ is the biological reactivity of Hg^0. The second term in Eq. 4 represents the mesophyll resistance, which in turn includes two parallel pathways to the extracellular water inside leaf stomata (Wesely, 1989). One pathway is through direct dissolution in the aqueous solution, while the other is reaction within the leaf. Due to the parallel nature of this term, the mesophyll resistance will be small if either H^* or f_0 is large.

The cuticular resistance is calculated as:

$$r_{lu}^{i} = \frac{r_{lu}^{i'}}{\frac{H_{\mathrm{Hg^0}}^{*}}{H_{\mathrm{SO_2}}^{*}} + f_0^{\mathrm{Hg^0}}} \qquad (5)$$

where $r_{lu}^{i'}$ is a reference cuticular resistance for the land type i which is scaled by the solubility and reactivity factors. The aerodynamic resistance in the lower part of the canopy is calculated as a function of the solar radiation (G):

$$r_{dc}^{i} = 100 \times \left(1 + \frac{1000}{G + 10}\right) \qquad (6)$$

The lower canopy surface resistance is calculated by scaling the resistances of the reference compounds sulfur dioxide (SO_2, which is soluble yet unreactive) and ozone (O_3, which is reactive yet insoluble) by the solubility and reactivity of Hg^0:

$$\frac{1}{r_{cl}^{i}} = \frac{H_{\mathrm{Hg^0}}^{*}}{H_{\mathrm{SO_2}}^{*}} \frac{1}{r_{cl,\mathrm{SO_2}}^{i}} + f_0^{\mathrm{Hg^0}} \frac{1}{r_{cl,\mathrm{O_3}}^{i}} \qquad (7)$$

The ground surface resistance is calculated in a similar manner as the lower canopy resistance:

$$\frac{1}{r_{gs}^{i}} = \frac{H_{\mathrm{Hg^0}}^{*}}{H_{\mathrm{SO_2}}^{*}} \frac{1}{r_{gs,\mathrm{SO_2}}^{i}} + f_0^{\mathrm{Hg^0}} \frac{1}{r_{gs,\mathrm{O_3}}^{i}} \qquad (8)$$

To account for reduced uptake by surfaces under cold temperatures (Wesely, 1989), an additional resistance (r_T) is calculated as a function of temperature:

$$r_T = 1000 \times \exp(-T - 4) \qquad (9)$$

This resistance is used to correct resistances for the ground surface, lower canopy, and cuticular surfaces, with the maximum effect capped to a factor of 2 times the original resistances (Jaeglé et al., 2018):

$$r^{i}_{gs,SO_2} = \min\left(r^{i^*}_{gs,SO_2} + r_T, 2r^{i^*}_{gs,SO_2}\right) \quad (10)$$

$$r^{i}_{gs,O_3} = \min\left(r^{i^*}_{gs,O_3} + r_T, 2r^{i^*}_{gs,O_3}\right) \quad (11)$$

$$r^{i}_{cl,SO_2} = \min\left(r^{i^*}_{cl,SO_2} + r_T, 2r^{i^*}_{cl,SO_2}\right) \quad (12)$$

$$r^{i}_{cl,O_3} = \min\left(r^{i^*}_{cl,O_3} + r_T, 2r^{i^*}_{cl,O_3}\right) \quad (13)$$

In addition to the temperature correction, the cuticular resistance is scaled by leaf area index:

$$r^{i'}_{lu} = \min\left(\frac{r^{i^*}_{lu}}{LAI^i} + r_T, \frac{2r^{i^*}_{lu}}{LAI^i}\right) \quad (14)$$

S2. Validation of offline dry deposition model with online results

The offline model (code can be found at: https://github.com/arifein/offline-drydep) for Hg^0 dry deposition velocities is compared with online GEOS-Chem simulations to validate its use in this study. Tests were made at different time resolutions of the offline model to identify a suitable time resolution that balances computational cost of the offline simulation with accuracy for predicting online simulated deposition velocities (Table S1). When using daily resolution of meteorological inputs, the mean error of the offline model over land grid cells is 15.2%. The large error illustrates the importance of accurately capturing the diurnal cycle of Hg^0 dry deposition. When the time resolution of the offline model is set to hourly, the mean error of land grid cells shrinks to 0.1%. Since we use two significant digits to report observed and simulated deposition velocities, we consider this error to be sufficiently small to apply the offline model in the current study. The offline model shows mean errors of similar relative magnitude (~0.1%) for $f_0 = 10^{-5}$ (Figure S1) and $f_0 = 1$ (Figure S2), and for January (Figure S1) and July (Figure S3).

Table S1. Mean error of offline model for monthly mean Hg^0 dry deposition velocities over land grid cells compared to online GEOS-Chem values for January and $f_0 = 1$. Different time resolutions of input meteorological parameters were tested.

**Time resolution of inputs**	**24 h**	**12 h**	**6 h**	**1 h**
Mean error (%)	15.2	10.7	5.0	0.1

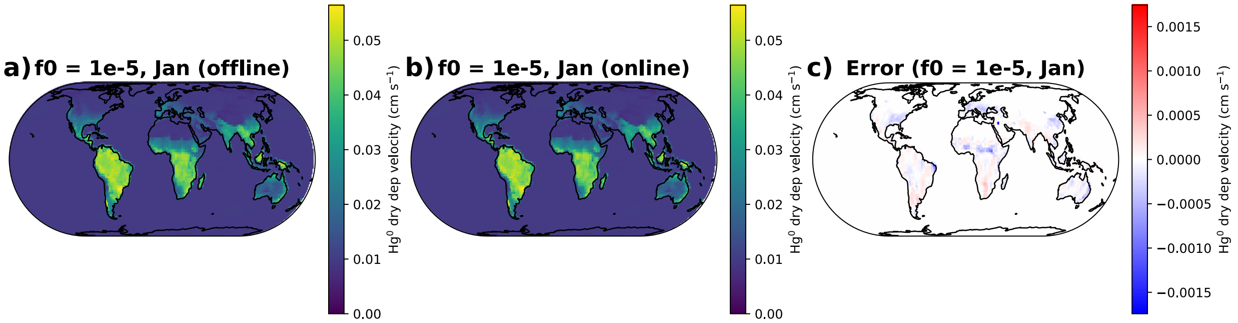

Figure S1. Comparing January monthly mean Hg^0 dry deposition velocities predicted by the (a) offline model for $f_0 = 10^{-5}$ (b) online GEOS-Chem model for $f_0 = 10^{-5}$. The absolute difference (offline – online) is shown in (c). Hourly time resolution was used for the offline model.

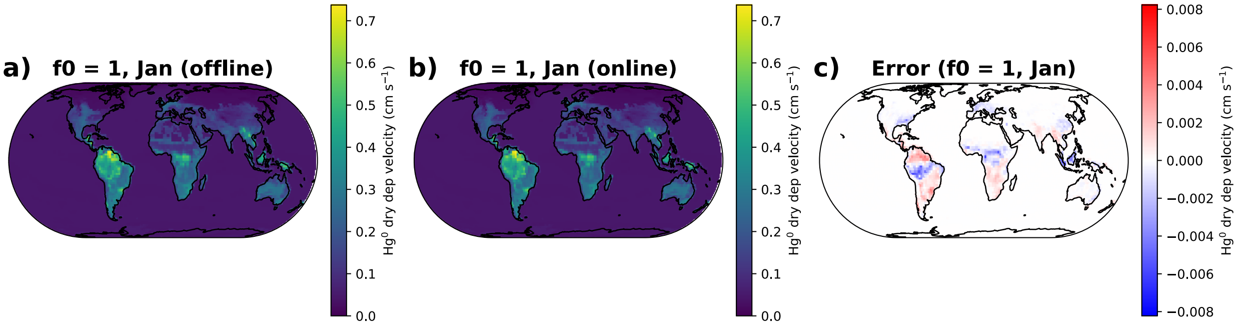

Figure S2. Comparing January monthly mean Hg^0 dry deposition velocities predicted by the (a) offline model for $f_0 = 1$ (b) online GEOS-Chem model for $f_0 = 1$. The absolute difference (offline – online) is shown in (c). Hourly time resolution was used for the offline model.

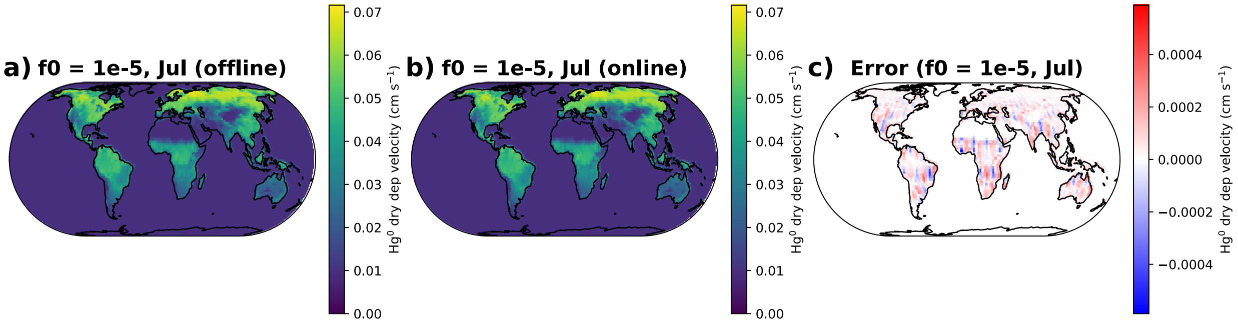

Figure S3. Comparing July monthly mean Hg^0 dry deposition velocities predicted by the (a) offline model for $f_0 = 10^{-5}$ (b) online GEOS-Chem model for $f_0 = 10^{-5}$. The absolute difference (offline – online) is shown in (c). Hourly time resolution was used for the offline model.

S3. Effect of land-surface adjustment on dry deposition evaluation

We follow the approach of Silva and Heald (2018) for ozone to account for the actual land category at the observation sites in the offline model calculations of Hg^0 dry deposition velocities. In the original model predictions, the model grid cell can contain multiple land cover types (e.g., ocean, deciduous forest, coniferous forest, grassland, etc.). Our adjusted predictions use only the observed land cover part of the grid cell to calculate the modelled dry deposition velocity. Figure S4 presents the comparison between original and adjusted dry deposition velocities at the litterfall stations in the compiled database. The largest adjustments (factor of ~3 increase) are in grid cells that are located in coastal areas: in Norton, Massachusetts, USA and Mt. Damei, Zhejiang, China. Since Hg^0 is relatively insoluble (0.11 M atm^{-1}), water-covered areas of grid cells show much lower Hg^0 dry deposition velocities than forested areas. Therefore, the adjustment to consider only the forested areas of the grid cell increases the Hg^0 dry deposition velocity in coastal grid cells. The land type adjustment therefore yields a fairer comparison between the model and a forested observation site.

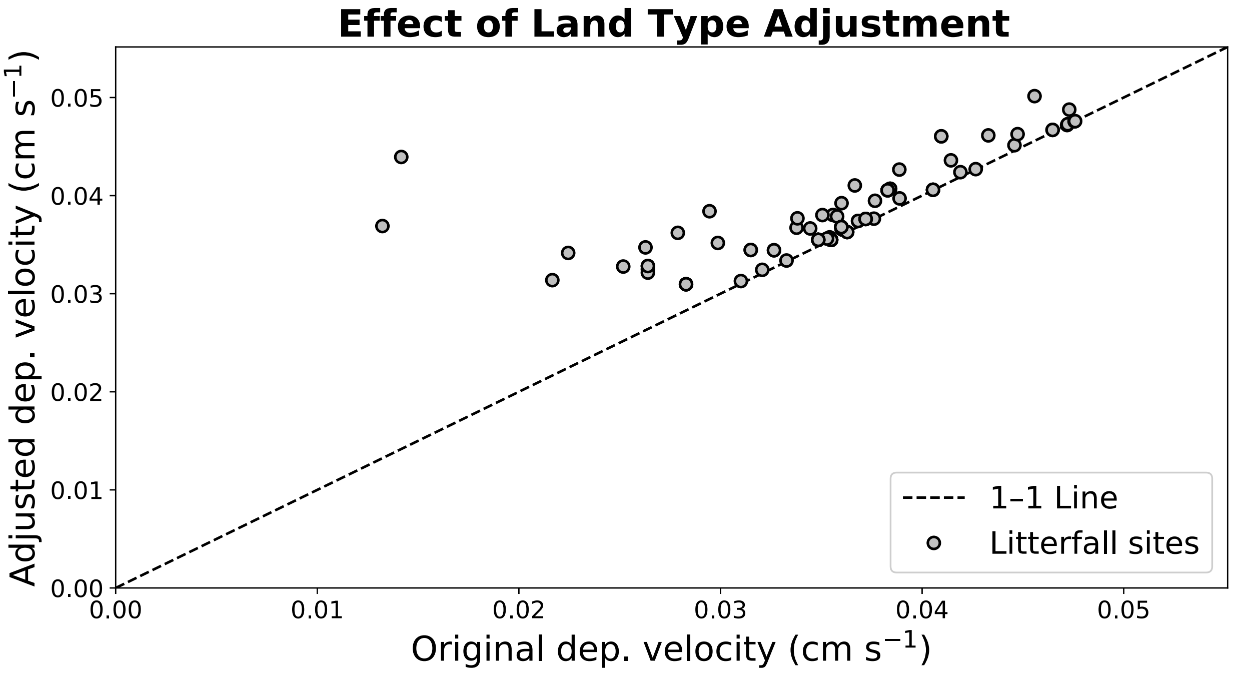

Figure S4. Illustrating the effect of the land type adjustment on predicted offline model Hg^0 dry deposition velocities for $f_0 = 10^{-5}$. Points show original model predictions for grid cells corresponding to litterfall measurement sites and adjusted model predictions when considering that 100% of the grid cell is covered by the observed forest type.

Figure S5 compares the original and adjusted model predictions with the litterfall measurements in the compiled database. Overall, the land type adjustment leads to more uniform model predictions, illustrating that much of the variability in the original model predictions for $f_0 = 10^{-5}$ is due to variability in the fraction of grid box covered by forest.

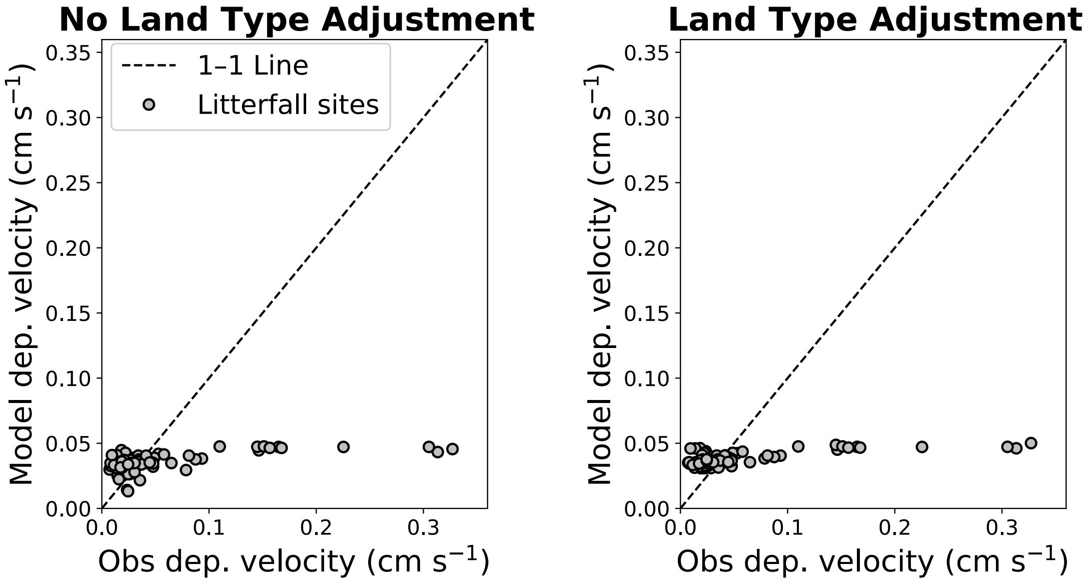

Figure S5. Comparison between measured litterfall deposition velocities and modelled Hg^0 dry deposition velocities, without and with land type adjustment for $f_0 = 10^{-5}$.

S4. Three-box model tuning of GEOS-Chem photoreduction

Due to its large uncertainty, the photoreduction rate of Hg^{2+} in organic aerosol and clouds is used as a tuning parameter in the GEOS-Chem Hg simulation (Horowitz et al., 2017; Shah et al., 2021). Upon changes to the simulation chemical mechanism or ocean setup, the reduction rate coefficient (α) is adjusted to give the best overall agreement with observed atmospheric Hg concentrations. Common practice has been to conduct GEOS-Chem Hg simulations with α adjusted ±10% until optimum agreement is achieved (http://wiki.seas.harvard.edu/geos-chem/index.php/Mercury#K_RED_JNO2). However, this approach requires multiple tuning runs of GEOS-Chem each time a parameter is changed within the Hg simulation.

To reduce the computational expense of the tuning procedure, we use a three-box model of the atmospheric Hg cycle written in Python (Figure S6, see the code repository at: https://github.com/arifein/atm-Hg-3boxmodel). The model includes Hg^0 and Hg^{2+} reservoirs in two hemispheric troposphere boxes and a global stratosphere box. Rate coefficients for the three-box model

are based on outputted GEOS-Chem fluxes and burdens, averaged over the two hemispheres in the troposphere and the stratosphere.

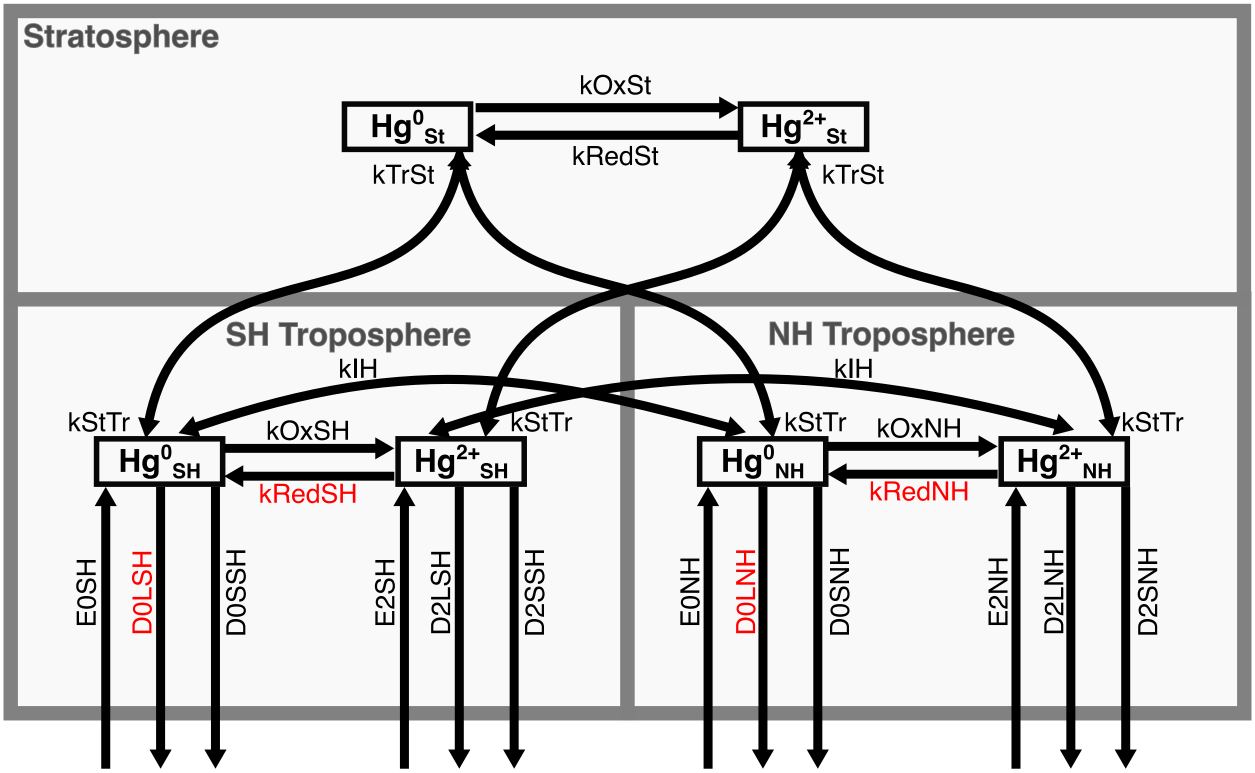

Figure S6. Diagram illustrating three-box model used to calibrate the reduction rate in GEOS-Chem simulations. Abbreviations in diagram are explained with the following — NH: Northern Hemisphere, SH: Southern Hemisphere, kOxSt: stratospheric oxidation, kRedSt: stratospheric reduction, kTrSt: troposphere to stratosphere exchange, kStTr: stratosphere to troposphere exchange, kIH: inter-hemispheric exchange, kOxSH: oxidation in SH troposphere, kRedSH: reduction in SH troposphere, E0SH: emissions of Hg^0 in SH, D0LSH: deposition of Hg^0 to land in SH, D0SSH: deposition of Hg^0 to sea in SH, E2SH: emissions of Hg^{2+} in SH, D2LSH: deposition of Hg^{2+} to land in SH, D2SSH: deposition of Hg^{2+} to sea in SH, kOxNH: oxidation in NH troposphere, kRedNH: reduction in NH troposphere, E0NH: emissions of Hg^0 in NH, D0LNH: deposition of Hg^0 to land in NH, D0SNH: deposition of Hg^0 to sea in NH, E2NH: emissions of Hg^{2+} in NH, D2LNH: deposition of Hg^{2+} to land in NH, D2SNH: deposition of Hg^{2+} to sea in NH. Highlighted in red are the fluxes that change significantly between GEOS-Chem simulations in this study: Hg^0 deposition to land and Hg^{2+} reduction fluxes.

We illustrate our GEOS-Chem tuning procedure through an example from the current study:

1) From the BASE simulation results, calculate the burdens and rate coefficients needed for the three-box model (Figure S6).
2) In the OBRIST simulation, the dry deposition of Hg^0 is enhanced, reducing the overall lifetime of Hg^0 and the burden. Thus, the Northern Hemisphere Hg^0 burden becomes too low compared to observations (Figure S9). We want the Northern Hemisphere Hg^0 burden to be roughly equivalent to BASE, which matches well with the annual mean of atmospheric Hg observations (Figure S9).
3) From the OBRIST simulation, we calculate the new rate coefficients of Hg^0 dry deposition to land (D0SSH and D0SNH) and implement these in the box model.
4) Using the box model, we run simulations at different tropospheric reduction rates of Hg^{2+} (Figure S7a). We identify the value of the tropospheric reduction rate (102 yr^{-1}) needed to yield the same burden of Hg^0 in the Northern Hemisphere as the BASE simulation. We focus on the Northern Hemisphere since the bulk of the atmospheric Hg measurements used to tune the model are located in the Northern Hemisphere (Figure S9).
5) The actual tuning parameter in GEOS-Chem is α, the reduction rate coefficient. Using three previous Hg runs in GEOS-Chem, we identify a logarithmic relationship between α and the reduction rate in the Northern Hemisphere (Figure S7b). Using the fitted logarithmic curve, we find required reduction rate (102 yr^{-1}) corresponds to $\alpha = 0.33$.
6) We run the OBRIST_R simulation with α set to 0.33.
7) We verified that the Northern Hemisphere tropospheric Hg^0 burden in the GEOS-Chem OBRIST_R simulation (1990 Mg) is roughly equivalent to the BASE simulation (1959 Mg). Also, we verified that the R^2 and bias of the OBRIST_R simulation compared to benchmark atmospheric Hg concentrations is similar to the BASE simulation (Figure S9).

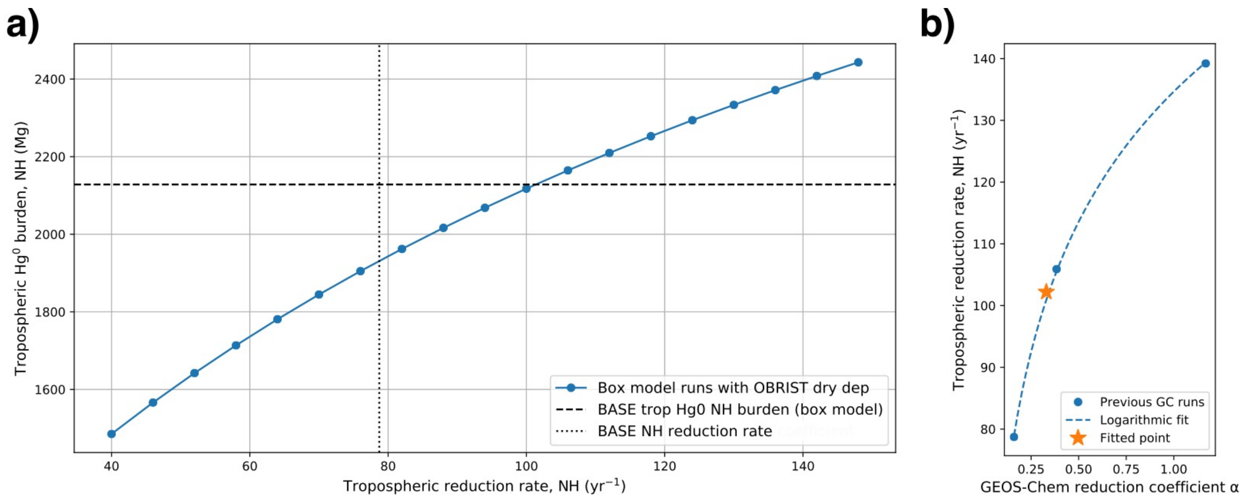

Figure S7. (a) The relationship between the box-modelled burden of Hg^0 in the Northern Hemisphere and the reduction rate of Hg^{2+} in the Northern Hemisphere troposphere, when using deposition rates corresponding to the OBRIST simulation. The BASE reduction rate and BASE Hg^0 burden in the Northern Hemisphere are shown with dotted and dashed lines, respectively. The intersection between the blue line and the dashed line indicates the required reduction rate in the Northern Hemisphere (102 yr^{-1}) to attain the same burden as BASE of Hg^0 in the Northern Hemisphere. (b) The relationship between the GEOS-Chem reduction coefficient, α, and the tropospheric reduction rate in the Northern Hemisphere. Using results from three previous runs of GEOS-Chem with different α parameters used, we fit a logarithmic relationship. This logarithmic relationship is used to calculate the value of α (0.33) that would yield the required rate of reduction in the Northern Hemisphere (102 yr^{-1}).

S5. Alternative solutions for matching Amazon dry deposition observations

The Hg^0 dry deposition velocity in the Amazon is underestimated by BASE and OBRIST simulations, which use a single parameter for the Hg^0 biological reactivity (f_0) in the dry deposition scheme (Section 3.1). We investigated whether the model also shows a bias in terms of ozone dry deposition velocities in the Amazon. In Figure S8, we compare the offline modelled ozone dry deposition velocities for 2015 with measurements in the Amazon from Fan et al. (1990) and Rummel et al. (2007), compiled in Silva and Heald (2018). The model underpredicts ozone dry deposition velocities by 39%, showing mean velocities of 0.49 cm s^{-1} compared to the observed 0.80 cm s^{-1}. For Hg^0, we compare model estimates using $f_0 = 3 \times 10^{-5}$ (OBRIST setting) with the mean of ten Amazon litterfall measurements compiled by Fostier et al. (2015). The mean modelled dry deposition velocity estimate is 0.098 cm s^{-1}, which is 53% below the mean measured value of 0.21 cm s^{-1}. Thus, the model appears to show a negative bias, of similar relative

magnitude, for both Hg^0 and ozone dry deposition in the Amazon. It is important to note, however, that only three measurement campaigns are available for the Amazon ozone dry deposition velocity estimate.

Given the similarity between both ozone and Hg^0 dry deposition biases, we explored resistance parameter solutions that could improve model-measurement agreement for both compounds. Initial tests of the GEOS-Chem parametrization identified two influential parameters for the ozone dry deposition velocity: the internal stomatal resistance (RI, called $r_{ix}^{i'}$ in Eq. 4) and the cuticular resistance (RLU, called r_{lu}^{i*} in Eq. 14). For the rainforest land class in GEOS-Chem, the parametrization uses RI = 200 and RLU = 1000. Figure S8 illustrates two scenarios that match the measured mean ozone dry deposition velocity: when RI = 65 or when RI = 100 and RLU = 500. When these parameter combinations are used to calculate the mean Hg^0 dry deposition velocities in the Amazon, the model predicts values of 0.12 and 0.11 cm s^{-1}, below the observed mean of 0.21 cm s^{-1}. By decreasing RI to 1, the Hg^0 dry deposition velocity (0.19 cm s^{-1}) approaches the observed value. However, the predicted ozone dry deposition velocity from this parameter scenario (2.1 cm s^{-1}) strongly overestimates the observed value (0.80 cm s^{-1}).

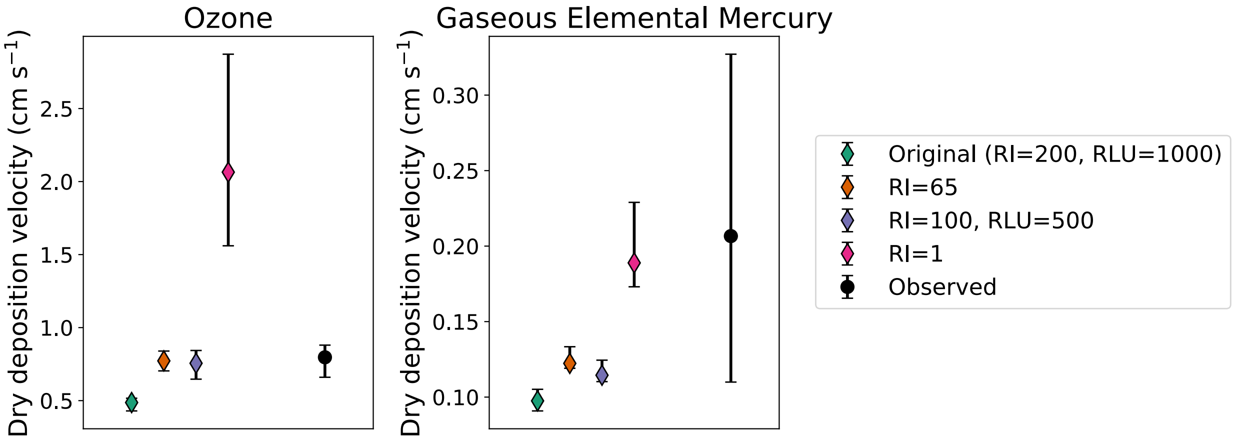

Figure S8. Comparing observed deposition velocities in the Amazon rainforest with offline model calculations using different estimates for internal stomatal resistance (RI) and cuticular resistance (RLU). Markers indicate mean value over observation locations and error bars indicate maximum and minimum values. Amazon ozone measurements are taken from Fan et al. (1990) and Rummel et al. (2007) and Amazon Hg litterfall measurements are compiled in Fostier et al. (2015). Elemental mercury dry deposition velocities are calculated assuming $f_0 = 3 \times 10^{-5}$.

In conclusion, although resistance parameters can be tuned to match the Hg^0 litterfall dry deposition velocities in the Amazon, this approach may yield unreasonable dry deposition velocities for other chemical compounds. Therefore, we proceeded with our approach of creating an additional

compound-specific parameter (f_0) for the Amazon rainforest region. The need for a new parameter may suggest that Amazon tree species take up Hg^0 at a higher rate than other trees, which would not be surprising given inter-species differences in Hg^0 uptake found in a European study (Wohlgemuth et al., 2022). Additional Hg^0 uptake measurements in the Amazon region would be needed to assess whether a species-specific vegetation uptake scheme could be applied in GEOS-Chem. Ongoing improvements to model dry deposition schemes (e.g., Clifton et al., 2020; Lin et al., 2019) may require future retuning of the Hg^0 biological reactivity to available vegetation uptake measurements.

S6. Additional model evaluation plots and table

Additional comparisons between simulations and observations are shown in Figure S9 for global surface TGM concentrations, Figure S10 for the overall global wet deposition comparison, Figure S11 for North American Hg wet deposition, and Figure S12 for European wet deposition. Table S2 lists the global Hg burdens and major fluxes for all simulations.

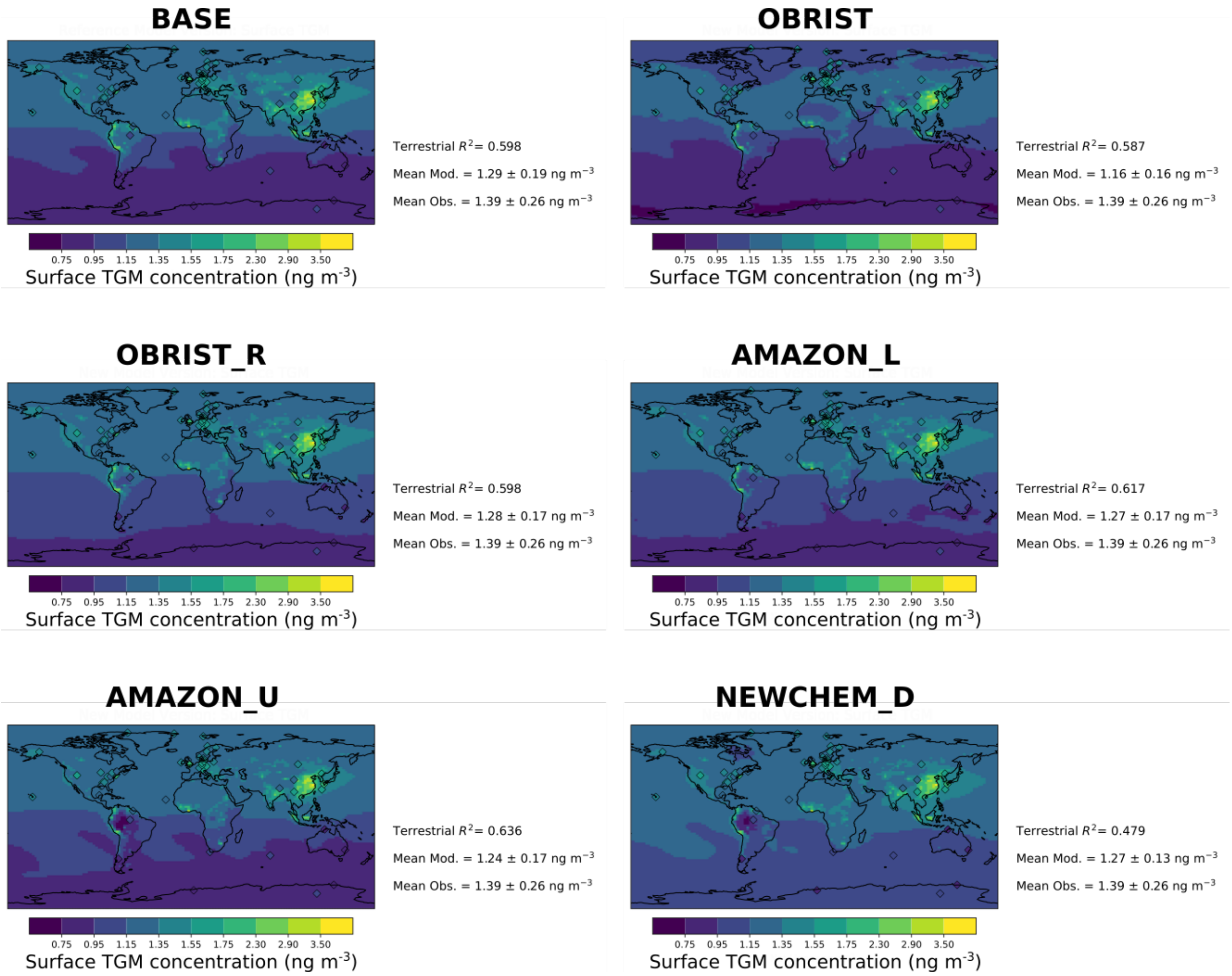

Figure S9. Comparing surface total gaseous mercury (TGM) concentrations from GEOS-Chem simulations with available observation stations. Measured atmospheric Hg concentrations are sourced from compilations (AMAP/UN Environment, 2019; Travnikov et al., 2017) and are courtesy of Hélène Angot. Model simulations are based on year 2015 and observations are averaged over 2013–2015. Statistics on right hand side of plots show the global summary of the coefficient of determination (R^2) and the mean and standard deviation of model and observations.

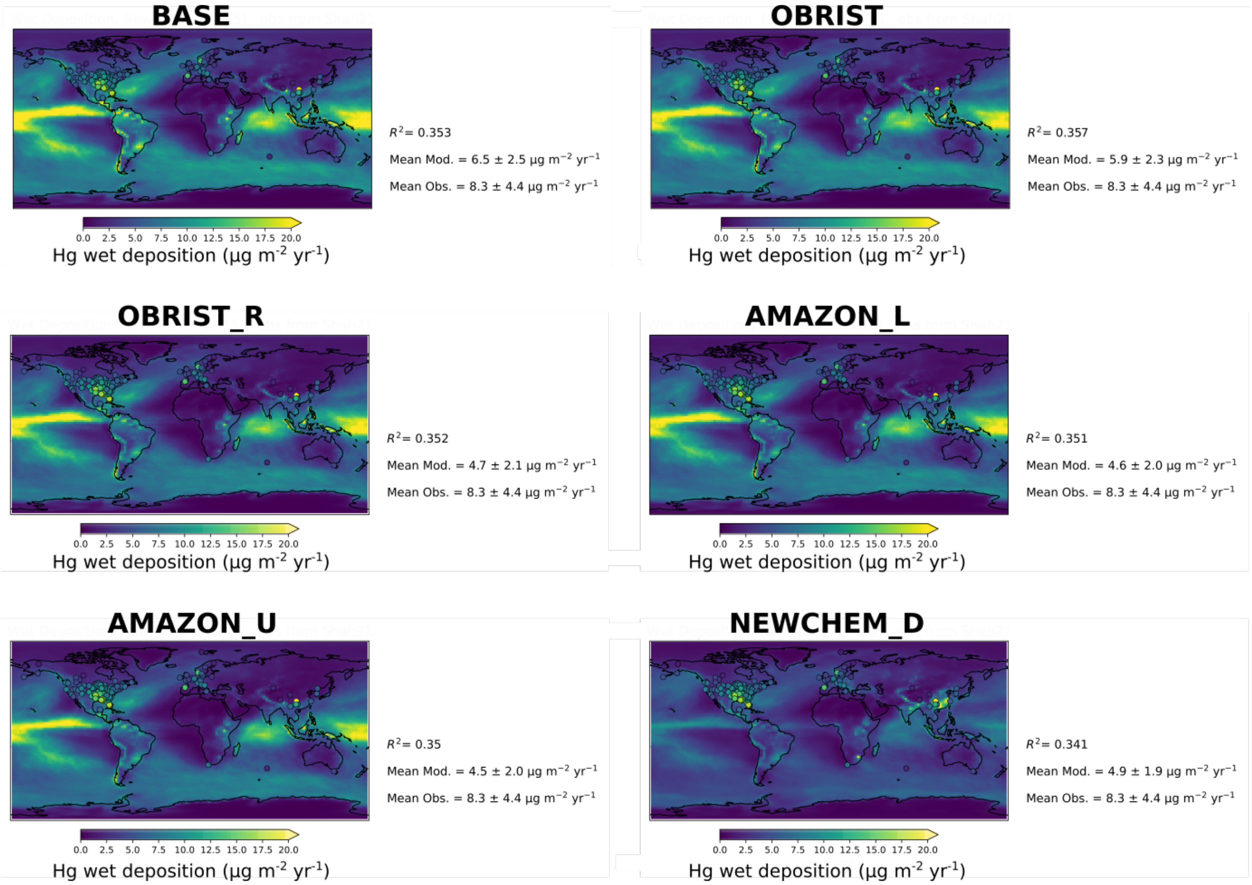

Figure S10. Comparing global Hg wet deposition fluxes from GEOS-Chem simulations with observations compiled by Shah et al. (2021) from measurements in Travnikov et al. (2017), Sprovieri et al. (2017), AMAP/UN Environment (2019), and Fu et al. (2016). Model simulations are for year 2015 and observations are for year 2010–2015. Statistics on right hand side of plots show the summary of the coefficient of determination (R^2) and the mean and standard deviation of model and observations. Due to site density in North America and Europe, additional plots are provided zoomed into those regions (Figures S11 and S12).

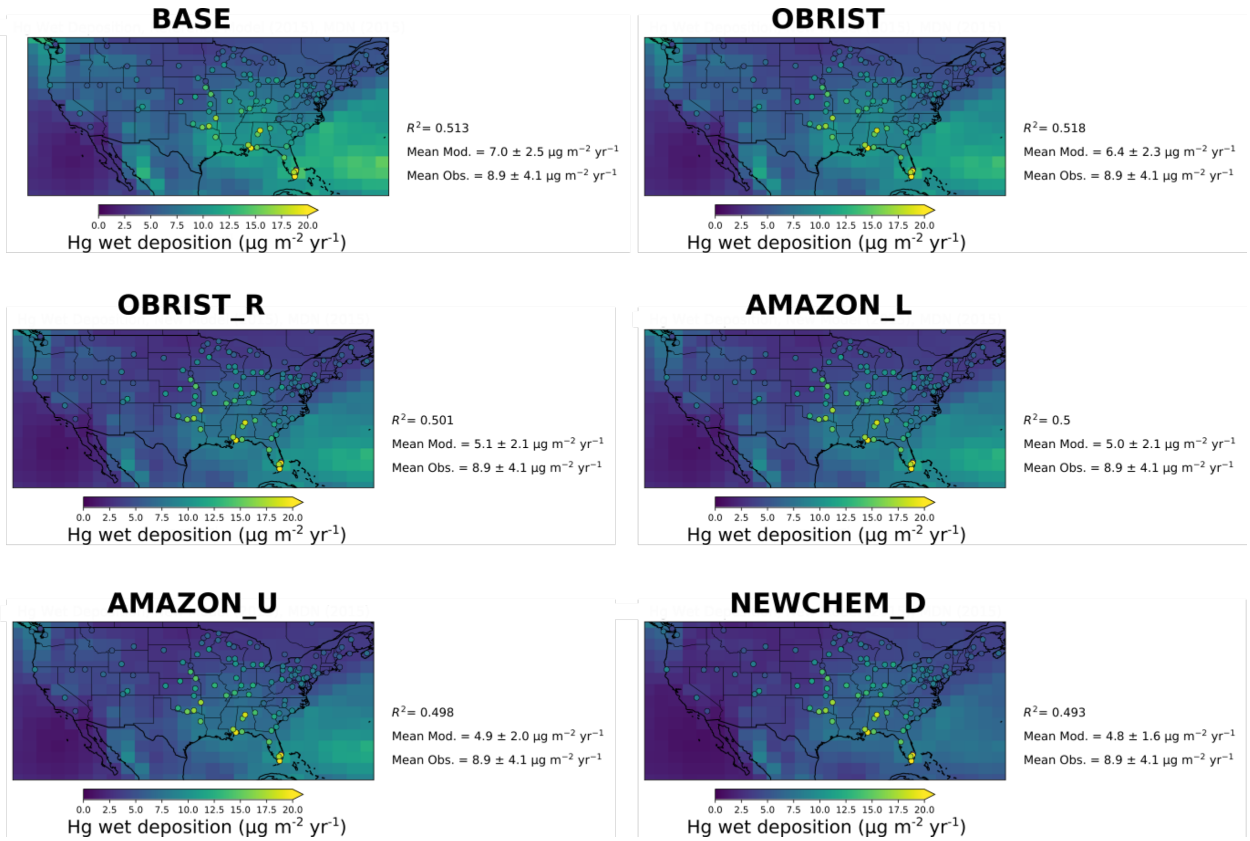

Figure S11. Comparing Hg wet deposition fluxes from GEOS-Chem simulations with available observation stations in continental USA from the Mercury Deposition Network (MDN, https://nadp.slh.wisc.edu/networks/mercury-deposition-network/). Observations were compiled Travnikov et al. (2017), courtesy of Hélène Angot. Model simulations and observations are both for the year 2015. Statistics on right hand side of plots show the summary of the coefficient of determination (R^2) and the mean and standard deviation of model and observations.

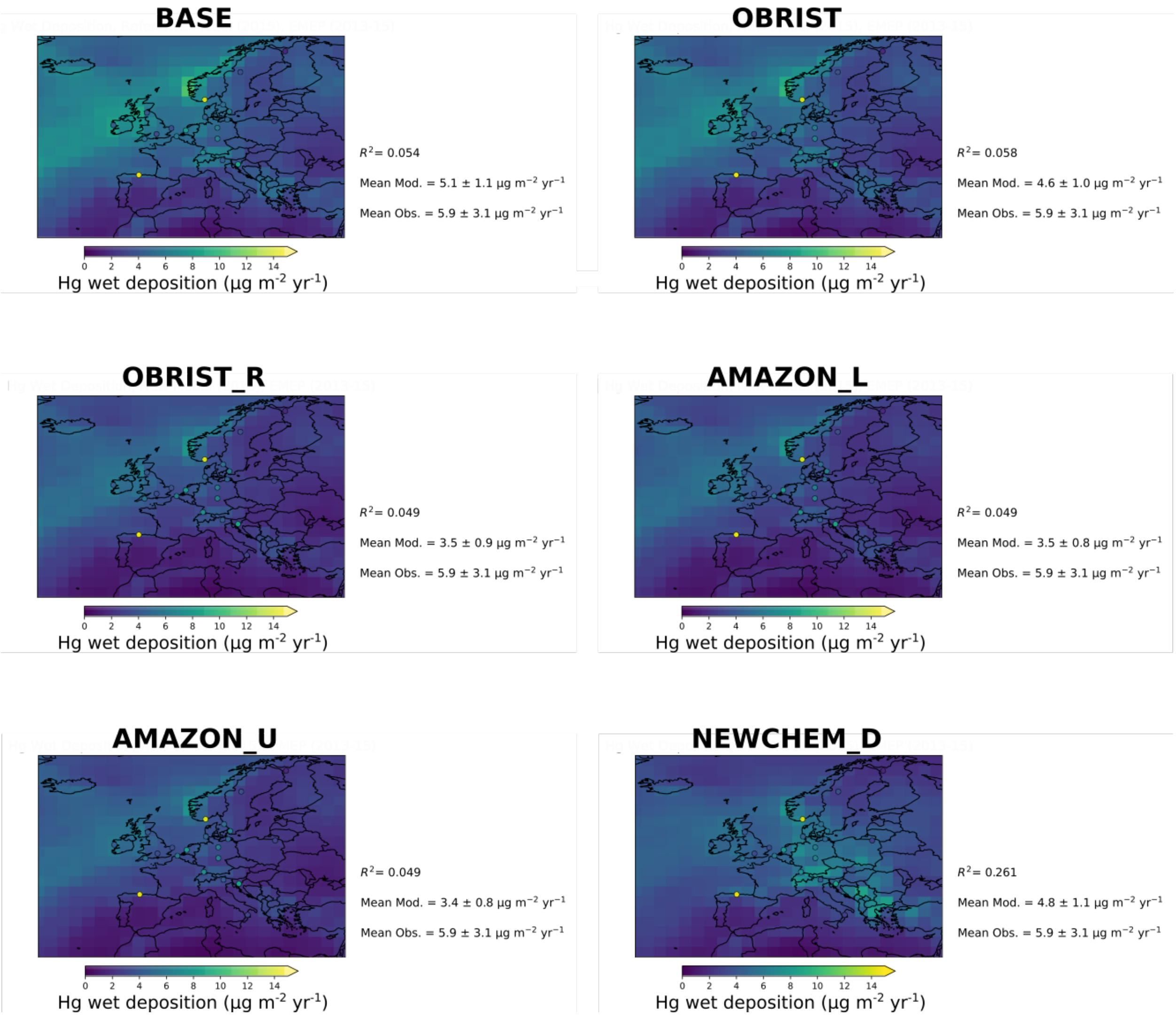

Figure S12. Comparing Hg wet deposition fluxes from GEOS-Chem simulations with available observation stations in Europe from the EMEP network (Tørseth et al., 2012). Observations were compiled Travnikov et al. (2017), courtesy of Hélène Angot. Model simulations are based on year 2015 and observations are averaged over 2013–2015. Statistics on right hand side of plots show the summary of the coefficient of determination (R^2) and the mean and standard deviation of model and observations.

Table S2. Global atmospheric Hg fluxes and burdens predicted by GEOS-Chem simulations.

**Simulation**	**Hg^0 burden (Mg)**	**Hg^{2+} burden (Mg)**	**Hg^0 land[a] emissions (Mg yr^{-1})**	**Hg^{2+} land[a] emissions (Mg yr^{-1})**	**Hg^0 net ocean emissions (Mg yr^{-1})**	**Hg^0 land deposition (Mg yr^{-1})**	**Hg^{2+} land deposition (Mg yr^{-1})**	**Hg^{2+} ocean deposition (Mg yr^{-1})**	**Hg^0 oxidation (Mg yr^{-1})**	**Hg^{2+} reduction (Mg yr^{-1})**
**BASE**	3649	812	3523	400	3225	1200	1069	4825	17675	12150
**OBRIST**	3348	776	3509	400	3343	1786	985	4471	16245	11175
**OBRIST_R**	3816	782	3493	400	3140	1961	824	4178	18527	13878
**AMAZON_L**	3717	775	3491	400	3162	2067	812	4112	18237	13669
**AMAZON_U**	3605	762	3486	400	3204	2276	790	3986	18160	13267
**NEWCHEM**	4770	392	3548	400	2903	1298	1199	4438	18752	13206
**NEWCHEM_D**	4492	342	3505	400	2996	2392	887	3770	17650	13075

[a] Refers to emissions from land areas, including the following sources: geogenic, soil, land re-emissions, snow, and anthropogenic activities

S7. Influence of meteorological year on modelled deposition

To test the sensitivity of our offline dry deposition results to the choice of year for the meteorological and LAI data, we ran the offline model under the AMAZON_U scenario using data from three different years (2014–2016). The median model predictions for the different datasets do not show substantial changes between the three meteorological years. Due to these sensitivity tests, we have used a single simulation year (2015), since comparable input data for leaf area input is not available over the entire observational time period. This result agrees with other studies that have determined GEOS-Chem modelled ozone dry deposition variability due to meteorology to be generally small (<5%, Silva and Heald, 2018). However, the simulated dry deposition variability has been found to be smaller than observed dry deposition variability, which could be due to the lack of moisture availability as a limiting factor in the dry deposition scheme (Clifton et al., 2020; Lin et al., 2019).

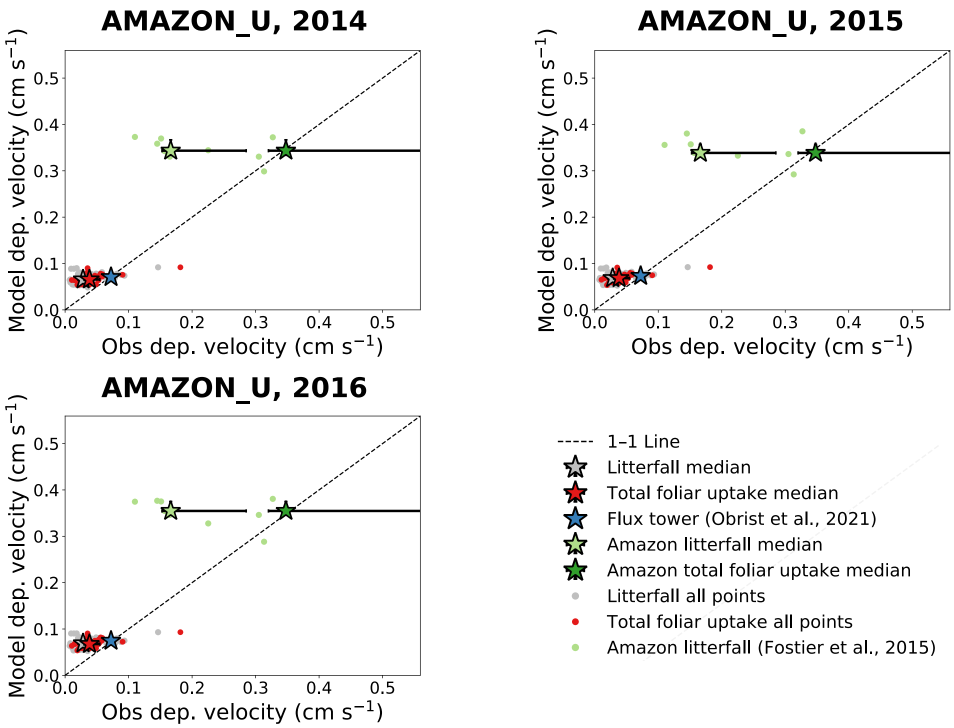

Figure S13. The impact of the meteorological and LAI year on the comparison between observed and simulated dry deposition velocities in the AMAZON_U case. Individual site measurements are indicated with filled circles and overall medians of measurement types are indicated with filled stars. Error bars show the interquartile range of measurements over different measurement locations. Model interquartile ranges are generally smaller than the size of the markers.

References

AMAP/UN Environment, 2019. Technical Background Report for the Global Mercury Assessment 2018. Arctic Monitoring and Assessment Programme, Oslo, Norway/UN Environment Programme, Chemicals and Health Branch, Geneva, Switzerland.

Clifton, O.E., Lombardozzi, D.L., Fiore, A.M., Paulot, F., Horowitz, L.W., 2020. Stomatal conductance influences interannual variability and long-term changes in regional cumulative plant uptake of ozone. Environ. Res. Lett. 15, 114059. https://doi.org/10.1088/1748-9326/abc3f1

Fan, S.-M., Wofsy, S.C., Bakwin, P.S., Jacob, D.J., Fitzjarrald, D.R., 1990. Atmosphere-biosphere exchange of CO_2 and O_3 in the central Amazon Forest. J. Geophys. Res. 95, 16851. https://doi.org/10.1029/JD095iD10p16851

Fostier, A.H., Melendez-Perez, J.J., Richter, L., 2015. Litter mercury deposition in the Amazonian rainforest. Environ. Pollut. 206, 605–610. https://doi.org/10.1016/j.envpol.2015.08.010

Fu, X., Yang, X., Lang, X., Zhou, J., Zhang, H., Yu, B., Yan, H., Lin, C.-J., Feng, X., 2016. Atmospheric wet and litterfall mercury deposition at urban and rural sites in China. Atmos. Chem. Phys. 16, 11547–11562. https://doi.org/10.5194/acp-16-11547-2016

Horowitz, H.M., Jacob, D.J., Zhang, Y., Dibble, T.S., Slemr, F., Amos, H.M., Schmidt, J.A., Corbitt, E.S., Marais, E.A., Sunderland, E.M., 2017. A new mechanism for atmospheric mercury redox chemistry: implications for the global mercury budget. Atmos. Chem. Phys. 17, 6353–6371. https://doi.org/10.5194/acp-17-6353-2017

Jaeglé, L., Shah, V., Thornton, J.A., Lopez-Hilfiker, F.D., Lee, B.H., McDuffie, E.E., Fibiger, D., Brown, S.S., Veres, P., Sparks, T.L., Ebben, C.J., Wooldridge, P.J., Kenagy, H.S., Cohen, R.C., Weinheimer, A.J., Campos, T.L., Montzka, D.D., Digangi, J.P., Wolfe, G.M., Hanisco, T., Schroder, J.C., Campuzano-Jost, P., Day, D.A., Jimenez, J.L., Sullivan, A.P., Guo, H., Weber, R.J., 2018. Nitrogen Oxides Emissions, Chemistry, Deposition, and Export Over the Northeast United States During the WINTER Aircraft Campaign. J. Geophys. Res. Atmos. 123. https://doi.org/10.1029/2018JD029133

Lin, M., Malyshev, S., Shevliakova, E., Paulot, F., Horowitz, L.W., Fares, S., Mikkelsen, T.N., Zhang, L., 2019. Sensitivity of Ozone Dry Deposition to Ecosystem-Atmosphere Interactions: A Critical Appraisal of Observations and Simulations. Global Biogeochem. Cycles 33, 1264–1288. https://doi.org/10.1029/2018GB006157

Rummel, U., Ammann, C., Kirkman, G.A., Moura, M.A.L., Foken, T., Andreae, M.O., Meixner, F.X., 2007. Seasonal variation of ozone deposition to a tropical rain forest in southwest Amazonia. Atmos. Chem. Phys. 21.

Shah, V., Jacob, D.J., Thackray, C.P., Wang, X., Sunderland, E.M., Dibble, T.S., Saiz-Lopez, A., Černušák, I., Kellö, V., Castro, P.J., Wu, R., Wang, C., 2021. Improved Mechanistic Model of the Atmospheric Redox Chemistry of Mercury. Environ. Sci. Technol. 55, 14445–14456. https://doi.org/10.1021/acs.est.1c03160

Silva, S.J., Heald, C.L., 2018. Investigating Dry Deposition of Ozone to Vegetation. J. Geophys. Res. Atmos. 123, 559–573. https://doi.org/10.1002/2017JD027278

Sprovieri, F., Pirrone, N., Bencardino, M., D'Amore, F., Angot, H., Barbante, C., Brunke, E.-G., Arcega-Cabrera, F., Cairns, W., Comero, S., Diéguez, M. del C., Dommergue, A., Ebinghaus, R., Feng, X.B., Fu, X., Garcia, P.E., Gawlik, B.M., Hageström, U., Hansson, K., Horvat, M., Kotnik, J., Labuschagne, C., Magand, O., Martin, L., Mashyanov, N., Mkololo, T., Munthe, J., Obolkin, V., Ramirez Islas, M., Sena, F., Somerset, V., Spandow, P., Vardè, M., Walters, C., Wängberg, I., Weigelt, A., Yang, X., Zhang, H., 2017. Five-year records of mercury wet deposition flux at GMOS sites in the Northern and Southern hemispheres. Atmos. Chem. Phys. 17, 2689–2708. https://doi.org/10.5194/acp-17-2689-2017

Tørseth, K., Aas, W., Breivik, K., Fjæraa, A.M., Fiebig, M., Hjellbrekke, A.G., Lund Myhre, C., Solberg, S., Yttri, K.E., 2012. Introduction to the European Monitoring and Evaluation Programme

(EMEP) and observed atmospheric composition change during 1972–2009. Atmos. Chem. Phys. 12, 5447–5481. https://doi.org/10.5194/acp-12-5447-2012

Travnikov, O., Angot, H., Artaxo, P., Bencardino, M., Bieser, J., D'Amore, F., Dastoor, A., De Simone, F., Diéguez, M. del C., Dommergue, A., Ebinghaus, R., Feng, X.B., Gencarelli, C.N., Hedgecock, I.M., Magand, O., Martin, L., Matthias, V., Mashyanov, N., Pirrone, N., Ramachandran, R., Read, K.A., Ryjkov, A., Selin, N.E., Sena, F., Song, S., Sprovieri, F., Wip, D., Wängberg, I., Yang, X., 2017. Multi-model study of mercury dispersion in the atmosphere: atmospheric processes and model evaluation. Atmos. Chem. Phys. 17, 5271–5295. https://doi.org/10.5194/acp-17-5271-2017

Wang, Y., Jacob, D.J., Logan, J.A., 1998. Global simulation of tropospheric O_3-NO_x-hydrocarbon chemistry: 1. Model formulation. J. Geophys. Res. 103, 10713–10725. https://doi.org/10.1029/98JD00158

Wesely, M.L., 1989. Parameterization of surface resistances to gaseous dry deposition in regional-scale numerical models. Atmos. Environ. 23, 1293–1304. https://doi.org/10.1016/0004-6981(89)90153-4

Wohlgemuth, L., Rautio, P., Ahrends, B., Russ, A., Vesterdal, L., Waldner, P., Timmermann, V., Eickenscheidt, N., Fürst, A., Greve, M., Roskams, P., Thimonier, A., Nicolas, M., Kowalska, A., Ingerslev, M., Merilä, P., Benham, S., Iacoban, C., Hoch, G., Alewell, C., Jiskra, M., 2022. Physiological and climate controls on foliar mercury uptake by European tree species. Biogeosciences 19, 1335–1353. https://doi.org/10.5194/bg-19-1335-2022
